# Quantitatively assessing the impact of the quality of SNOMED CT subtype hierarchy on cohort queries

**DOI:** 10.1093/jamia/ocae272

**Published:** 2024-11-09

**Authors:** Xubing Hao, Xiaojin Li, Yan Huang, Jay Shi, Rashmie Abeysinghe, Cui Tao, Kirk Roberts, Guo-Qiang Zhang, Licong Cui

**Affiliations:** McWilliams School of Biomedical Informatics, The University of Texas Health Science Center at Houston, Houston, TX 77030, United States; Department of Neurology, McGovern Medical School, The University of Texas Health Science Center at Houston, Houston, TX 77030, United States; Department of Neurology, McGovern Medical School, The University of Texas Health Science Center at Houston, Houston, TX 77030, United States; Intermountain Healthcare, Denver, CO 80218, United States; Department of Neurology, McGovern Medical School, The University of Texas Health Science Center at Houston, Houston, TX 77030, United States; Department of Artificial Intelligence and Informatics, Mayo Clinic, Jacksonville, FL 32224, United States; McWilliams School of Biomedical Informatics, The University of Texas Health Science Center at Houston, Houston, TX 77030, United States; McWilliams School of Biomedical Informatics, The University of Texas Health Science Center at Houston, Houston, TX 77030, United States; Department of Neurology, McGovern Medical School, The University of Texas Health Science Center at Houston, Houston, TX 77030, United States; McWilliams School of Biomedical Informatics, The University of Texas Health Science Center at Houston, Houston, TX 77030, United States

**Keywords:** SNOMED CT, quality impact, quantitative assessment, cohort query, EHR data

## Abstract

**Objective:**

SNOMED CT provides a standardized terminology for clinical concepts, allowing cohort queries over heterogeneous clinical data including Electronic Health Records (EHRs). While it is intuitive that missing and inaccurate subtype (or *is-a*) relations in SNOMED CT reduce the recall and precision of cohort queries, the extent of these impacts has not been formally assessed. This study fills this gap by developing quantitative metrics to measure these impacts and performing statistical analysis on their significance.

**Material and Methods:**

We used the Optum de-identified COVID-19 Electronic Health Record dataset. We defined micro-averaged and macro-averaged recall and precision metrics to assess the impact of missing and inaccurate *is-a* relations on cohort queries. Both practical and simulated analyses were performed. Practical analyses involved 407 missing and 48 inaccurate *is-a* relations confirmed by domain experts, with statistical testing using Wilcoxon signed-rank tests. Simulated analyses used two random sets of 400 *is-a* relations to simulate missing and inaccurate *is-a* relations.

**Results:**

Wilcoxon signed-rank tests from both practical and simulated analyses (*P*-values < .001) showed that missing *is-a* relations significantly reduced the micro- and macro-averaged recall, and inaccurate *is-a* relations significantly reduced the micro- and macro-averaged precision.

**Discussion:**

The introduced impact metrics can assist SNOMED CT maintainers in prioritizing critical hierarchical defects for quality enhancement. These metrics are generally applicable for assessing the quality impact of a terminology’s subtype hierarchy on its cohort query applications.

**Conclusion:**

Our results indicate a significant impact of missing and inaccurate *is-a* relations in SNOMED CT on the recall and precision of cohort queries. Our work highlights the importance of high-quality terminology hierarchy for cohort queries over EHR data and provides valuable insights for prioritizing quality improvements of SNOMED CT's hierarchy.

## Introduction

SNOMED CT is globally recognized as the most comprehensive and multilingual clinical healthcare terminology.^1^ It encompasses a broad spectrum of concepts including clinical findings, procedures, body structures, and other healthcare-related terms. Its hierarchical structure, built on subtype or *is-a* relations, allows for the classification of concepts at various levels of granularity, making it a value resource for clinical documentation, research, and decision support.

SNOMED CT provides a standardized and structured terminology for representing clinical concepts and facilitating data exchange and integration across diverse health information systems, including Electronic Health Records (EHRs). For example, the Observational Health Data Sciences and Informatics (OHDSI), the largest observational data network worldwide, has adopted SNOMED CT as a standard terminology for diagnoses (or conditions) in the Observational Medical Outcomes Partnership (OMOP) Common Data Model (CDM); and source data coded in other vocabularies such as the International Classification of Diseases, Ninth Revision, Clinical Modification (ICD-9-CM) and the International Classification of Diseases, Tenth Revision, Clinical Modification (ICD-10-CM) are mapped to SNOMED CT codes.^2^

As a common reference terminology to which other code systems can be mapped, SNOMED CT not only allows clinical queries over heterogeneous data^3^ but also has been widely used to facilitate the retrieval and identification of patient cohorts for retrospective observational studies.^4^^,^^5^ For example, SNOMED CT concept hierarchies have been utilized to construct and define shareable clinical conditions (or value sets) for identifying patients within an EHR system and across clinically integrated networks.^6^ Effective support for patient cohort queries with SNOMED CT relies heavily on its subtype (or *is-a*) hierarchy, the taxonomic backbone of the terminology. Hence, maintaining the quality of the SNOMED CT subtype hierarchy is paramount. Considerable efforts have been spent in developing computational approaches to ensure the quality of the SNOMED CT subtype hierarchy.^7–12^ For example, Bodenreider introduced a lexical-based approach aimed at uncovering missing *is-a* relations in SNOMED CT.^7^ This approach uses the names of SNOMED CT concepts to construct logical definitions for the concepts, uses these logical definitions to infer a separate subtype hierarchy, and compares it with the original SNOMED CT subtype hierarchy to identify missing *is-a* relations between concepts. In our previous works,^8^^,^^9^ we devised a hybrid approach by systematically analyzing lexical features of concepts within non-lattice subgraphs in SNOMED CT to detect missing *is-a* relations. Liu et al^10^^,^^11^ have explored convolutional neural networks and transfer learning techniques to place new concepts in the SNOMED CT hierarchy given that the new concept’s name is known. In a recent study, we developed a deep learning-based approach that utilizes concept name features, hierarchical structure, and logical definitions to identify missing *is-a* relations between concept pairs exhibiting a containment lexical pattern in SNOMED CT.^12^

Although considerable efforts have been spent in developing computational approaches to identify quality defects of the SNOMED CT subtype hierarchy, no prior study has focused on quantitatively evaluating the direct impact of its hierarchy quality on patient cohort queries. While it is intuitive that missing *is-a* relations reduce the recall and inaccurate *is-a* relations lower the precision of cohort queries, the degree of these impacts has not been formally demonstrated previously. In this study, we address this gap by defining quantitative metrics to measure these impacts, enabling further statistical analysis of their significance. Such a systematic approach to assessing the impact of hierarchical defects can inform the prioritization of quality improvements of SNOMED CT, ensuring that the most critical issues are addressed first, ultimately enhancing its overall quality and usability.

##  

The goal of this study is to assess, in a quantitative manner, how the quality of the SNOMED CT subtype hierarchy directly influences the effectiveness of patient cohort queries. To achieve this, we use the Optum de-identified COVID-19 Electronic Health Record dataset to measure the extent to which the inaccurate *is-a* relations and missing *is-a* relations in SNOMED CT impact the precision and recall of patient cohort queries, respectively. To the best of our knowledge, our work is the first that attempts a quantitative evaluation of the SNOMED CT hierarchy quality’s impact on the results of actual cohort queries.

## Materials and methods

The EHR dataset against which patient cohort queries are conducted is the Optum de-identified COVID-19 Electronic Health Record dataset licensed by The University of Texas Health Science Center at Houston. Our primary focus in this study is using the patient diagnosis data in the Optum COVID-19 data to evaluate how the quality of SNOMED CT's hierarchy affects cohort queries. Since the patient diagnosis data was coded in three vocabularies (SNOMED CT, ICD-9-CM, and ICD-10-CM), we extracted concept mappings between ICD-9-CM/ICD-10-CM and SNOMED CT from the OHDSI Standardized Vocabularies^2^ to support cohort queries. We further gathered two sets of hierarchical defects in the SNOMED CT (September 2023 US Edition): a set comprising missing *is-a* relations and another set comprising inaccurate *is-a* relations, which were validated by domain experts in our previous works for quality assurance of SNOMED CT’s subtype hierarchy. We then defined micro-averaged and macro-averaged metrics to quantitatively measure the overall impact of missing *is-a* relations and inaccurate *is-a* relations for cohort queries, and performed practical and simulated impact analyses.

### Optum de-identified COVID-19 electronic health record dataset

The Optum COVID-19 data was derived from the electronic health records of over 700 hospitals and 7000 clinics across the United States.^13^ It consists of longitudinal clinical records for approximately 7 million unique individuals who have documented clinical care with a documented diagnosis of COVID-19 or acute respiratory illness after 02/01/2020 and/or documented COVID-19 testing regardless of their results.^14^ This dataset contains patient-level information including demographics, diagnoses, procedures, lab tests, care settings, medications prescribed or administered, and mortality. The data was de-identified by independent statistical experts in accordance with Health Insurance Portability and Accountability Act (HIPAA) statistical de-identification rules and are managed in accordance with the Optum customer data use agreement.^13^ This dataset has been used for retrospective analyses of COVID-19 outcomes for various health conditions, where patient cohort identification is always involved in the very first step of the analysis.^14–16^

In this work, we mainly utilized the patient diagnosis data with 567 441 527 records in the Optum COVID-19 data to measure the impact of the SNOMED CT’s hierarchy quality on cohort queries. We loaded the Optum COVID-19 data into a MongoDB database with a novel indexing strategy called Event-level Inverted Index (ELII)^17^ that supports fast query execution on clinical events for patient cohort identification. Previous evaluation of the performance of ELII demonstrates that ELII achieved an average speed acceleration of 1037.6 times on classical (non-temporal) queries compared to the baseline approach without using ELII.^17^ The efficiency provided by ELII is critical in processing such a large dataset, enabling us to perform extensive and timely analyses. It is essential not just for faster query execution, but for ensuring the scalability and robustness of our analyses.

### ICD-9-CM/ICD-10-CM to SNOMED CT concept mappings

Each record in the diagnosis data in the Optum COVID-19 data was coded in either SNOMED CT, ICD-9-CM, or ICD-10-CM. Since our goal is to assess the hierarchy quality impact of SNOMED CT, we used the concept mappings between ICD-9-CM/ICD-10-CM and SNOMED CT from the OHDSI Standardized Vocabularies (v5.0 version) to support cohort queries. The OHDSI Standardized Vocabularies contain concept mappings from over 130 source vocabularies,^2^ where SNOMED CT serves as a standard terminology for diagnoses (or conditions) and other vocabularies such as ICD-9-CM and ICD-10-CM are mapped to SNOMED CT standard concepts. Mappings between ICD-9-CM/ICD-10-CM and SNOMED CT are crucial for ensuring comprehensive retrieval of patient cohorts. Consider the example of the ICD-10-CM code “*A06: Amebiasis*,” which maps to the SNOMED CT concept “*111910009: Amebic infection (disorder)*.” By using mappings, a query on the SNOMED CT concept would also capture patients diagnosed with the ICD-10-CM code, preventing the omission of relevant cases.

### SNOMED CT hierarchical defects

Two main issues in SNOMED CT's subtype hierarchy that can affect the accuracy of cohort queries are: missing *is-a* relations and inaccurate *is-a* relations. Missing *is-a* relations occur when a necessary *is-a* relation between two concepts is absent, meaning a concept should be classified as a subtype of another concept but is not. In contrast, inaccurate *is-a* relations arise when an existing *is-a* relation between two concepts is incorrectly represented, meaning a concept is wrongly placed as a subtype of another concept. Missing *is-a* relations may lead to the exclusion of relevant patients from query results, thereby reducing recall. On the other hand, incorrect is-a relations may cause irrelevant patients to be included in query results, resulting in decreased precision. To assess their real-world impact on cohort queries over the Optum COVID-19 data, we used our previous works to compile a set of missing *is-a* relations and another set of inaccurate *is-a* relations in SNOMED CT, which were validated by domain experts.

We aggregated missing *is-a* relations that were manually verified by domain experts in six of our previous studies.^8^^,^^9^^,^^12^^,^^18–20^ In each study, potential missing *is-a* relations were identified using our automated detection method, which generated a large pool of candidates. From this pool, random samples were selected for manual expert evaluation. The evaluation was conducted by one or two domain experts. These experts included clinical terminology specialists from the National Library of Medicine (NLM) and physician experts with extensive experience in evaluating clinical terminologies. When two experts were involved, only those missing *is-a* relations that were agreed upon by both experts were considered valid. Table 1 shows a brief summary of the detection method used in each study, the number and type of domain expert(s) involved in the evaluation, the number of valid missing *is-a* relations, as well as the SNOMED CT version used.

**Table 1.** Summary information of previous studies for identifying missing *is-a* relations.

Previous work	Method	No. and type of domain expert(s)	SNOMED CT version	No. of valid missing *is-a* relations
Hao et al^18^	Substring replacement approach using lexical features of concept names and existing *is-a* relations	1 physician expert	March 2022	89
Abeysinghe et al^12^	Deep learning-based approach utilizing concept names, hierarchical structure, and logical definitions	1 NLM expert, 1 physician expert	September 2019	216
Zheng et al^20^	Transformation-based approach replacing noun chunks with candidates more general than the noun chunk	1 physician expert	September 2019	173
Cui et al^9^	Combining non-lattice subgraphs and enriched lexical attributes of concepts	1 NLM expert, 1 physician expert	September 2017	185
Zheng et al^19^	Exhaustive detection using words and noun phrases in concepts and performing subset inclusion	1 physician expert	September 2017	90
Cui et al^8^	Identifying lexical patterns in non-lattice subgraphs indicative of errors	2 NLM experts	September 2015	59

In total, 812 missing *is-a* relations were aggregated from these six previous studies. However, these studies used earlier versions of SNOMED CT to identify missing *is-a* relations, and some of the missing *is-a* relations have been rectified in the September 2023 version of SNOMED CT. Therefore, if the two concepts involved in a missing *is-a* relation were active in the September 2023 version and linked by a direct or indirect *is-a* relation between the two concepts, we removed them from our set of missing *is-a* relations. As a result, 368 out of 812 were removed. For instance, Abeysinghe et al^12^ identified a missing *is-a* relation between “*Phlebitis of the anterior tibial vein (disorder)*” and “*Phlebitis of tibial vein (disorder)*” in the September 2019 version of SNOMED CT, which was confirmed by domain experts. However, in the September 2023 version of SNOMED CT, “*Phlebitis of tibial vein (disorder)*” is a parent of “*Phlebitis of the anterior tibial vein (disorder)*.” This previously identified missing *is-a* relation was fixed in the newer version and therefore removed from our list of missing *is-a* relations.

For the set of inaccurate *is-a* relations, we used the inaccurate *is-a* relations in the *Clinical finding* and *Procedure* subhierarchies of SNOMED CT (March 2022 version) identified by our recent study.^21^ This study presented an automated approach using concept lexical features to suggest inaccurate *is-a* relations. One physician expert conducted the manual evaluation and confirmed a total of 67 inaccurate *is-a* relations. We further removed the relations that have been rectified in the September 2023 version of SNOMED CT, resulting in 48 inaccurate *is-a* relations from this version.

Given that the set of inaccurate *is-a* relations come from *Clinical finding* and *Procedure* subhierarchies, we further focused our attention on these two subhierarchies for the missing *is-a* relations to ensure consistency. This resulted in a final set of 407 missing *is-a* relations.

### Quantitative metrics for assessing the impact of missing and inaccurate *is-a* relations

In this study, we focus on assessing the impact of missing and inaccurate *is-a* relations on atomic cohort queries. An atomic cohort query is defined as “Identifying patients diagnosed with *c*,” where *c* is a concept in the SNOMED CT, such as “Identifying patients diagnosed with *primary malignant neoplasm of kidney*.” Note that the query result for “Identifying patients diagnosed with *c*” will include patients diagnosed with concept *c* or any of its descendant concepts.

When a missing *is-a* relation occurs between two concepts *c_1_* and *c_2_* (ie, *c_1_ is-a c_2_* is missing in the terminology structure), patients diagnosed with *c_1_* and its descendants will be missing from the result of the cohort query “Identifying patients diagnosed with *p*,” where *p* is an ancestor of *c_2_* or *c_2_* itself. In other words, the missing *is-a* connection between *c_1_* and *c_2_* reduces the recall for all atomic cohort queries involving *c_2_* and its ancestors. Formally, given a concept *c*, we denote the set of patients diagnosed as *c* and its descendants by *N_c_* (ie, the query result of “Identifying patients diagnosed with *c*”). For any ancestor *p* of concept *c_2_*, the cohort query for *p* has a recall of |$N_{p}|/|N_{c_{1}}\cup N_{p}$*|*. Since our objective is to measure the overall impact of a missing *is-a* relation on cohort identification, we define micro-averaged recall and macro-averaged recall (inspired by Tsoumakas et al^22^) as


$$\frac{\sum_{p\in c_{2}^{↑}}\left|N_{p}\right|}{\sum_{p\in c_{2}^{↑}}{|N_{c_{1}}\cup \;N}_{p}|}\;\mathrm{and}\;\frac{1}{\left|c_{2}^{↑}\right|}\times \sum_{p\in c_{2}^{↑}\;}\frac{{|N}_{p}|}{\left|N_{c_{1}}\cup \;N_{p}\right|}$$

respectively, where $c_{2}^{↑}$ denotes the set of concepts consisting of *c_2_* and its ancestors.

Note that macro-averaged recall first calculates the recall for each concept in $c_{2}^{↑}$ individually and then take the average of these individual recall values. Micro-averaged recall, on the other hand, computes the overall recall by summing the sizes of patient sets $N_{p}$ for all concepts in $c_{2}^{↑}$ and dividing this by the sum of the sizes of patient sets ${N_{c_{1}}\cup \;N}_{p}$ for all concepts in $c_{2}^{↑}$. In other words, macro-averaged recall treats each concept equally by averaging individual recalls across concepts, while micro-averaged recall aggregates patient data across all concepts and calculates recall as if performing a single cohort query.

When an inaccurate *is-a* relation occurs between concepts *c_1_* and *c_2_* (ie, *c_1_ is-a c_2_* is an inaccurate relation), patients diagnosed with *c_1_* and its descendants will be inaccurately included in the result of the cohort query “Identifying patients diagnosed with *p*” where *p* is an ancestor of *c_2_* or *c_2_* itself. In other words, the inaccurate *is-a* connection between *c_1_* and *c_2_* decreases the precision for all atomic cohort queries involving *c_2_* and its ancestors. More formally, for any ancestor *p* of concept *c_2_*, the cohort query for *p* has a precision of |$N_{p}{-N}_{c_{1}}|/|N_{p}|$. Similarly, we define micro-averaged precision and macro-averaged precision as


$$\frac{\sum_{p\in c_{2}^{↑}}\left|N_{p}-N_{c_{1}}\right|}{\sum_{p\in c_{2}^{↑}}{|N}_{p}|}\;\mathrm{and}\;\frac{1}{\left|c_{2}^{↑}\right|}\times \sum_{p\in c_{2}^{↑}\;}\frac{{|N}_{p}-N_{c_{1}}|}{\left|N_{p}\right|}$$

respectively to assess the overall impact of an inaccurate *is-a* relation on cohort identification.

### Impact analyses of missing and inaccurate *is-a* relations

We analyzed the quality impact of missing and inaccurate *is-a* relations on cohort queries in two means: practical and simulated.

For the practical analysis, we computed the micro- and macro-averaged recall for the set of 407 validated missing *is-a* relations as well as micro- and macro-averaged precision for the set of 48 validated inaccurate *is-a* relations. Based on these metrics, we further performed statistical analyses to test our hypotheses that missing *is-a* relations would reduce the recall of cohort queries and inaccurate *is-a* relations would reduce the precision of cohort queries.

Regarding the simulated analysis, we randomly selected 400 *is-a* relations in SNOMED CT to simulate missing *is-a* relations and another 400 to simulate inaccurate *is-a* relations. Note that with a confidence level of 95% and a margin of error of 5%, 384 relations would be sufficient for a population of 370 014 SNOMED CT concepts. We calculated the micro- and macro-averaged recall for the simulated missing *is-a* relations and micro- and macro-averaged precision for the simulated inaccurate *is-a* relations, and then performed the hypotheses testing.

## Results

### Summary of concept mappings

In this work, we used OHDSI Standardized Vocabularies (v5.0 version) to obtain the concept mappings between ICD-9-CM/ICD-10-CM and SNOMED CT for supporting cohort queries, where the vocabularies versions used are ICD-9-CM Version 32, ICD-10-CM FY2024, and 2023-09-01 SNOMED CT US Edition. There are a total of 370 014 concepts from SNOMED CT, 99 130 codes from ICD-10-CM, and 17 564 codes from ICD-9-CM. Regarding concept mappings, 97 126 ICD-10-CM codes are mapped to standard SNOMED CT concepts, and 17 268 ICD-9-CM codes are mapped to standard SNOMED CT concepts.

### Quantitative assessment metrics for missing and inaccurate *is-a* relations

The micro- and macro-averaged recall for the validated set of 407 missing *is-a* relations can be found in the Supplementary Materials (see missing-relations-metrics.pdf). Table 2 shows ten examples of the missing *is-a* relations, where for each missing *is-a* relation between subconcept *c_1_* and superconcept *c_2_*, we demonstrate the number of patients retrieved when performing the cohort query for *c_2_* (ie, |$N_{c_{2}}|$), the number of patients that should be retrieved if the missing *is-a* relation were present in SNOMED CT (ie, *|*$N_{c_{1}}\cup N_{c_{2}}$*|*), the recall of the cohort query for *c_2_* (ie, |$N_{c_{2}}|/|N_{c_{1}}\cup N_{c_{2}}$*|*), as well as the micro-averaged recall and macro-averaged recall for the missing *is-a* relation.

**Table 2.** Ten examples of missing *is-a* relations and their quantitative impact metrics.

Subconcept *c_1_*	Superconcept *c_2_*	|$N_{c_{2}}|$	*|* $N_{c_{1}}\cup N_{c_{2}}$ *|*	$\frac{\left|N_{c_{2}}\right|}{\left|N_{c_{1}}\cup N_{c_{2}}\right|}$	Micro-averaged recall	Macro-averaged recall
Lesion of joint (finding)	Finding of lesion (finding)	2165	1 605 587	0.0013	0.9046	0.6671
Spondylosis (disorder)	Arthritis of spine (disorder)	197 609	945 118	0.2091	0.9724	0.9261
Traumatic blister of trunk, infected (disorder)	Traumatic blister (disorder)	29 808	29 885	0.9974	0.9999	0.9996
Cerebellar ataxic gait (finding)	Cerebellar gait (finding)	33 246	33 249	0.9999	1.0000	0.9999
Toxic metabolic encephalopathy (disorder)	Toxic encephalopathy (disorder)	67 908	192 904	0.3520	0.9978	0.9537
Osteoarthritis of knee (disorder)	Arthritis of knee (disorder)	47 552	660 907	0.0719	0.9668	0.8733
Abrasion of pelvic region (disorder)	Superficial injury of pelvic region (disorder)	4	4616	0.0009	0.9999	0.9444
Cognitive communication disorder (disorder)	Cognitive disorder (disorder)	27 482	42 301	0.6497	0.9997	0.9779
Inflammatory carcinoma of breast (disorder)	Inflammatory disorder of breast (disorder)	60 238	60 272	0.9994	0.9999	0.9999
Gastric ulcer without hemorrhage AND without perforation (disorder)	Peptic ulcer without hemorrhage AND without perforation (disorder)	24 122	100 349	0.2404	0.9988	0.9494

Take the missing *is-a* relation between “*Osteoarthritis of knee (disorder)*” and “*Arthritis of knee (disorder)*” as an example. There are 47 552 patients retrieved when performing the cohort query for “*Arthritis of knee (disorder)*” against the EHR dataset. If this missing *is-a* relation were present in SNOMED CT, the number of patients retrieved would be 660 907. Therefore, the recall of the cohort query for “*Arthritis of knee (disorder)*” is 0.0719 (=47 552/660 907). The overall impact metrics of this missing *is-a* relation (micro- and macro-averaged recall), which considers both “*Arthritis of knee (disorder)*” and its ancestors, are 0.9668 and 0.8733, respectively.

The micro- and macro-averaged precision for the validated set of 48 inaccurate *is-a* relations can be found in the Supplementary Materials (see inaccurate-relations-metrics.pdf). Note that 16 out of 48 relations were marked as “NA” since no patients were retrieved when performing cohort queries for the subconcept and superconcept.

Table 3 shows ten examples of the inaccurate *is-a* relations, where for each inaccurate *is-a* relation between subconcept *c*_1_ and superconcept *c_2_*, we demonstrate the number of patients retrieved when performing the cohort query for *c_2_* (ie, $\left|N_{c_{2}}\right|$), the number of patients that should be retrieved if the inaccurate relation were absent from SNOMED CT (ie, |$N_{c_{2}}{-N}_{c_{1}}|$), the precision of the cohort query for *c_2_* (ie, |$N_{c_{2}}{-N}_{c_{1}}|/|N_{c_{2}}|$), as well as the micro-averaged precision and macro-averaged precision for the inaccurate *is-a* relation.

**Table 3.** Ten examples of inaccurate *is-a* relations and their quantitative impact metrics.

Subconcept *c_1_*	Superconcept *c_2_*	|$N_{c_{2}}|$	|$N_{c_{2}}{-N}_{c_{1}}|$	$\frac{\left|N_{c_{2}}{-N}_{c_{1}}\right|}{\left|N_{c_{2}}\right|}$	Micro-averaged precision	Macro-averaged precision
Finding of thought content (finding)	Disturbance in content of thought (finding)	212 681	8	0.0000	0.9599	0.5358
Metabolic encephalopathy (disorder)	Toxic metabolic encephalopathy (disorder)	192 904	68 056	0.3528	0.9974	0.9494
Language finding (finding)	Speech finding (finding)	397 503	321 009	0.8076	0.9951	0.9359
Tracheobronchial finding (finding)	Finding of trachea (finding)	155 451	51 197	0.3293	0.9937	0.8624
Closed injury of head (disorder)	Closed wound of head (disorder)	201 061	193 926	0.9645	0.9997	0.9969
Toxic encephalopathy (disorder)	Toxic metabolic encephalopathy (disorder)	192 904	153 319	0.7948	0.9988	0.9833
Anorectal fistula (disorder)	Rectal fistula (disorder)	4580	2767	0.6041	0.9999	0.9829
Arteriovenous anastomosis (procedure)	Arterial anastomosis (procedure)	1202	1177	0.9792	0.9999	0.9934
Dissection of cerebral artery (disorder)	Dissecting aneurysm of cerebral artery (disorder)	345	5	0.0145	0.9999	0.9615
Procedure on shoulder joint (procedure)	Procedure on joint of shoulder girdle (procedure)	436	71	0.1628	0.9999	0.8290

Take the example of inaccurate *is-a* relation between “*Toxic encephalopathy (disorder)*” and “*Toxic metabolic encephalopathy (disorder)*” as an example. There are 192 904 patients retrieved when performing the cohort query for “*Toxic metabolic encephalopathy (disorder)*” against the EHR dataset. If this inaccurate *is-a* relation were absent from SNOMED CT, the number of patients retrieved would be 153 319. Therefore, the precision of the cohort query for “*Toxic metabolic encephalopathy (disorder)*” is 0.7948 (=153 319/192 904). The overall impact metrics of this inaccurate *is-a* relation (micro- and macro-averaged precision) are 0.9988 and 0.9833, respectively.

### Practical impact analyses

We used the validated sets of 407 missing *is-a* relations and 48 inaccurate *is-a* relations for the practical impact analysis. Due to the non-normal distribution of our data, we used the Wilcoxon signed-rank test (one-sided).^23^

For the impact of missing *is-a* relations on the recall of cohort queries, we performed Wilcoxon signed-rank tests regarding both micro-averaged recall and macro-averaged recall. Our null hypothesis is that the median micro-averaged/macro-averaged recall for missing *is-a* relations is equal to 1, whereas our alternative hypothesis is that it is significantly <1. The resulting *P*-values are both <.001 (8.143119e−28 regarding the median micro-averaged recall and 8.144609e−28 regarding the median macro-averaged recall). Therefore, we conclude that there is sufficient evidence to reject the null hypothesis and accept the alternative hypothesis that the median micro-averaged recall and median macro-averaged recall for missing *is-a* relations are significantly <1. This indicates the significant impact of missing *is-a* relations on the recall of cohort queries.

Regarding the impact of inaccurate *is-a* relations on the precision of cohort queries, our null hypothesis is that the median micro-averaged/macro-averaged precision is equal to 1, while our alternative hypothesis is that it is significantly <1. The resulting *P*-values of the Wilcoxon signed-rank tests are both <.001 (1.862645e−09 regarding the median micro-averaged precision and 1.862645e−09 regarding the median macro-averaged precision). Therefore, we conclude that there is sufficient evidence to reject the null hypothesis and accept the alternative hypothesis that the median micro-averaged precision and median macro-averaged precision for inaccurate *is-a* relations are significantly <1. This indicates a significant impact of inaccurate *is-a* relations on the precision of cohort queries.

### Simulated impact analyses

We also performed impact analyses based on the 400 simulated missing *is-a* relations and 400 simulated inaccurate *is-a* relations that were randomly selected. For simulated missing *is-a* relations, the *P*-values of the Wilcoxon signed-rank tests for the median micro-averaged recall and median macro-averaged recall are both <.001 (2.229618e−23 and 2.229841e−23, respectively). Regarding simulated inaccurate *is-a* relations, the *P*-values for the Wilcoxon signed-rank tests for the median micro-averaged precision and median macro-averaged precision are both <.001 (4.264624e−19 and 4.265069e−19, respectively). Consequently, we conclude that there is sufficient evidence to reject the null hypotheses and accept the alternative hypotheses, indicating a significant impact of missing and inaccurate *is-a* relations on cohort queries. These findings are consistent with our earlier conclusions drawn from the practical impact analyses based on the actual missing and inaccurate *is-a* relations.

## Discussion

In this paper, we used a de-identified EHR dataset to assess the impact of hierarchical defects (ie, missing and inaccurate *is-a* relations) in SNOMED CT on patient cohort queries over the EHR dataset. We defined quantitative metrics (micro-averaged and macro-averaged recall and precision) to measure the overall impact of missing and inaccurate *is-a* relations on cohort queries. Both practical analyses, using two validated sets of missing and inaccurate *is-a* relations, and simulated analyses, using two randomly selected sets of *is-a* relations, demonstrated a significant impact of missing and accurate *is-a* relations on the recall and precision of cohort queries, respectively.

### Direct and overall quantitative impact of hierarchical defects

Note that the micro- and macro-averaged metrics that we defined are intended to measure the overall or global impact of a hierarchical defect (ie, a missing *is-a* relation or inaccurate *is-a* relation between a subconcept and a superconcept). This was achieved by considering not only the direct impact on the recall/precision of the cohort query for the superconcept but also the indirect impact on the recall/precision of the cohort queries for all ancestors of the superconcept. In other words, the overall impact of the problematic relation is obtained by aggregating its direct impact on the superconcept’s cohort query and indirect impact on the cohort queries for the superconcept’s ancestors.

For our practical impact analyses, we used 407 missing and 48 inaccurate *is-a* relations aggregated from our prior works and validated by domain experts. While these numbers may appear small compared to the total 370 014 SNOMED CT concepts, it is crucial to note that these represent validated instances from focused efforts, not the entire universe of hierarchical defects in SNOMED CT. For example, in our previous work,^18^ we identified 3228 potential missing *is-a* relations, and domain expert evaluation of a random sample of 100 revealed 89 of them are indeed missing *is-a* relations. This suggests that a larger number of missing or inaccurate relations likely exists, but evaluating all of them requires extensive manual review, which is time-consuming and labor-intensive. In other words, the actual scale of hierarchical defects is likely much larger than the 407 (or 48) relations that we directly analyzed.

### Practical applications and relevance

This work enables researchers to gain insights into how missing and inaccurate *is-a* relations in SNOMED CT affect cohort queries, allowing them to formulate mitigation strategies (eg, a quality check of the code sets used for queries) that enhance the accuracy and reliability of their results. In addition, the impact metrics developed in this work can guide developers and maintainers of SNOMED CT in prioritizing which hierarchical defects need urgent attention, ultimately leading to a more robust and reliable terminology. Enhanced precision and recall in cohort queries can facilitate more effective epidemiological studies, clinical trials, and population health management, making this work crucial for advancing the utility of EHR data in real-world applications. The impact metrics can also be generally applicable for evaluating any terminology with a subtype hierarchy and its associated cohort query applications that rely on this terminology as a knowledge source.

### Mappings between terminologies

Ideally, there would be no need for mapping between terminologies like SNOMED CT and ICD, as having a unified standard would eliminate data loss during conversion. However, given the widespread use of both terminologies in different contexts, this is not yet feasible. SNOMED CT uses a polyhierarchical structure allowing for more flexible and detailed representation of concepts and relationships between concepts, making it well-suited for clinical documentation and decision support. ICD codes follow a monohierarchical (tree-like) structure, making it more streamlined for administrative purposes like statistical reporting and billing. For cohort queries, SNOMED CT is generally considered to be better suited due to its finer granularity and flexibility, which allow for more precise identification and analysis of patient groups. While the use of mappings introduces some complexity, it is necessary to integrate diverse coding systems and avoid missing relevant patient data.

While other mapping sources, such as the Centers for Medicare & Medicaid Services (CMS)/NLM mappings and the Unified Medical Language System (UMLS) synonymous mappings, are available, we selected the OHDSI mappings for this study due to their specific focus on supporting cohort identification and large-scale observational research. OHDSI mappings are continuously curated and updated by the OHDSI vocabulary team to ensure alignment with research-driven use cases, whereas CMS/NLM mappings are primarily intended for administrative and billing purposes. The OHDSI team integrates mappings from multiple sources, including UMLS, or develops them de novo when necessary.^2^ Furthermore, the OHDSI community actively contributes to improving the quality of these mappings by reporting and addressing issues as they arise, ensuring that they remain robust for research applications. By using the OHDSI mappings, we benefit from this ongoing refinement and the broader community effort to ensure the quality of mappings.

### Recognizing and mitigating potential terminology-related bias in EHR-based research

Researchers use EHR data to generate new knowledge aiming at enhancing healthcare for individuals and populations. Gaps in clinical terminologies and their hierarchies used in EHR, such as missing or inaccurate *is-a* relations in SNOMED CT, can introduce biases that may affect the validity of research findings. When these gaps lead to the exclusion of important data subsets, the risk of biased conclusions increases, as valuable data may be unintentionally overlooked.

To ensure the integrity of studies relying on EHR data, it is essential that researchers are fully aware of the potential biases inherent in the terminologies used. Such awareness is critical to prevent unintentional data omissions and misinterpretation of results. To mitigate potential biases, researchers could consider validating their findings through alternative data resources. Moreover, clearly acknowledging and discussing these potential biases in research publications allows the broader research community to interpret the results with a fuller understanding of the limitations.

In addition, terminology like SNOMED CT is frequently updated with the SNOMED CT US Edition released biannually and the International Edition updated monthly. These updates reflect ongoing quality improvements, corrections, and content expansions, which can introduce challenges when using multiple versions in large-scale population health studies. Limiting a study to a single version of SNOMED CT, preferably the most recent version available, may help mitigate the risk of bias. The latest version incorporates the most accurate, up-to-date concept definitions and relations, reducing the likelihood of outdated or missing information impacting study results. However, for scenarios where studies need to include data spanning multiple versions, especially in longitudinal research or when working with historical EHR datasets, researchers should carefully document the versions used and consider conduct sensitivity analyses to evaluate how changes across versions may influence study outcomes. This can help identify if and how the use of multiple versions introduces bias.

In this study, we demonstrated the tangible impact that missing or inaccurate *is-a* relations can have on the recall and precision of cohort queries. By quantifying these effects, our work offers researchers a foundation for recognizing and mitigating the inherent biases in the terminologies used in the EHR data. As EHR data play an increasingly prominent role in health research, this work underscores the need for ongoing efforts to improve the accuracy and completeness of clinical terminologies.

### Limitations and future work

A limitation of our work is that we only assessed the impact of missing or inaccurate *is-a* relations on atomic cohort queries, which target single SNOMED CT concepts. The impact on cohort queries involving multiple SNOMED CT concepts and Boolean operators requires further investigation.

In addition, while using SNOMED CT codes for performing cohort queries over the EHR dataset, we may encounter situations where a concept in ICD-9-CM or ICD-10-CM does not have a matching concept in SNOMED CT. In such cases, we only retained patient data with matching SNOMED CT codes based on the concept mappings obtained from the OHDSI Standardized Vocabularies. Thus, the resulting database only comprised a subset of the original dataset with matching SNOMED CT codes, and our impact analyses were performed based on this subset.

## Conclusion

In this work, we introduced quantitative metrics to measure the overall impact of missing *is-a* relations and inaccurate *is-a* relations in SNOMED CT on the recall and precision of cohort queries over a de-identified EHR dataset. We conducted both practical and simulated impact analyses. For the practical analyses, we collected a set of 407 missing *is-a* relations and another set of 48 inaccurate *is-a* relations in SNOMED CT confirmed by domain experts, obtained their micro- and macro-averaged recall and precision for cohort queries targeting individual SNOMED CT concepts, and performed the Wilcoxon signed-rank tests. For the simulated analyses, we randomly selected two sets of *is-a* relations to mimic missing and inaccurate *is-a* relations. Both practical and simulated analyses showed a significant impact of missing and inaccurate *is-a* relations on the recall and precision of cohort queries, highlighting the importance of high-quality biomedical terminologies for patient cohort queries over EHR data.

## Supplementary Material

ocae272_Supplementary_Data

## Data Availability

The data underlying this article are described in the article and in its online supplementary material.
