# Supplementary material for: Quantitatively assessing the impact of the quality of SNOMED CT subtype hierarchy on cohort queries
Source: J Am Med Inform Assoc. 2024 Nov 9;32(1):89–96. doi: 10.1093/jamia/ocae272 (PMC11648736; doi:10.1093/jamia/ocae272)
Supplement: ocae272_Supplementary_Data [file ocae272_supplementary_data.zip › Suppl/missing-relations-metrics.pdf]

| subconcept id | subconcept | superconcept id | superconcept | micro-averaged recall | macro-averaged recall |
|---|---|---|---|---|---|
| 770754006 | 2p21 microdeletion syndrome without cystinuria (disorder) | 719652007 | 2p21 microdeletion syndrome (disorder) | 1 | 1 |
| 283060001 | Abrasion of pelvic region (disorder) | 283037009 | Superficial injury of pelvic region (disorder) | 0.999883896 | 0.944426271 |
| 111275004 | Abscess of nasal septum (disorder) | 363173007 | Inflammatory disorder of cartilage (disorder) | 0.999998758 | 0.999991426 |
| 301561000119108 | Abscess of tendon sheath of muscle of left forearm (disorder) | 202988003 | Abscess of tendon of forearm (disorder) | 1 | 1 |
| 723880004 | Abscess of upper respiratory tract (disorder) | 129134004 | Inflammatory disorder of upper respiratory tract (disorder) | 0.999897218 | 0.999862011 |
| 290235009 | Accidental diclofenac poisoning (disorder) | 216483006 | Accidental poisoning caused by antirheumatic (disorder) | 1 | 1 |
| 216519004 | Accidental poisoning caused by phenothiazine-based tranquilizer (disorder) | 48534006 | Poisoning caused by phenothiazine-based tranquilizer (disorder) | 0.999999522 | 0.997236891 |
| 15714481000119100 | Acquired genu recurvatum of right knee (disorder) | 46101007 | Acquired genu recurvatum (disorder) | 1 | 1 |
| 111261006 | Acquired musculoskeletal deformity of upper arm (disorder) | 40668007 | Acquired musculoskeletal deformity (disorder) | 0.999925258 | 0.998993535 |

| | | | | | |
|---|---|---|---|---|---|
| 308111000119106 | Acquired pes planus of right foot (disorder) | 203534009 | Acquired pes planus (disorder) | 0.999995994 | 0.999641754 |
| 323481000119109 | Acquired varus deformity of left ankle (disorder) | 15741881000119100 | Acquired deformity of left ankle (disorder) | 0.999999846 | 0.999995887 |
| 202479004 | Acromioclavicular joint pain (finding) | 267949000 | Shoulder joint pain (finding) | 0.999999772 | 0.999999121 |
| 229262002 | Active pelvic movements (regime/therapy) | 229261009 | Active trunk movements (regime/therapy) | 1 | 1 |
| 342181000119107 | Acute dacryoadenitis of left eye (disorder) | 127343008 | Acute disease of eye (disorder) | 1 | 1 |
| 336571000119100 | Acute dacryoadenitis of right eye (disorder) | 127343008 | Acute disease of eye (disorder) | 1 | 1 |
| 132601000119102 | Acute deep venous thrombosis of pelvic vein (disorder) | 128055005 | Deep venous thrombosis of pelvic vein (disorder) | 0.999999991 | 0.999940462 |
| 222008 | Acute epiglottitis with obstruction (disorder) | 408669002 | Acute laryngitis with obstruction (disorder) | 0.999998458 | 0.999832963 |
| 191572009 | Acute exacerbation of chronic schizoaffective schizophrenia (disorder) | 191531007 | Acute exacerbation of chronic schizophrenia (disorder) | 0.999899694 | 0.929051295 |
| 90628007 | Acute gastric ulcer with perforation but without obstruction (disorder) | 34921009 | Acute peptic ulcer with perforation but without obstruction (disorder) | 0.999990148 | 0.955704894 |

| | | | | | |
|---|---|---|---|---|---|
| 66673003 | Acute gastrojejunal ulcer with hemorrhage AND with perforation but without obstruction (disorder) | 47064007 | Acute peptic ulcer with hemorrhage AND with perforation but without obstruction (disorder) | 0.999999526 | 0.996762598 |
| 59515005 | Acute gastrojejunal ulcer with hemorrhage but without obstruction (disorder) | 22157005 | Acute peptic ulcer with hemorrhage but without obstruction (disorder) | 0.999998231 | 0.992192994 |
| 30514008 | Acute gastrojejunal ulcer without hemorrhage AND without perforation (disorder) | 67964002 | Acute gastric ulcer without hemorrhage AND without perforation (disorder) | 0.99998089 | 0.996699447 |
| 59867002 | Acute osteomyelitis of multiple sites (disorder) | 7127005 | Osteitis of multiple sites (disorder) | 0.999993514 | 0.999145131 |
| 58085004 | Acute peptic ulcer without hemorrhage AND without perforation but with obstruction (disorder) | 45485004 | Acute peptic ulcer without hemorrhage AND without perforation (disorder) | 1 | 1 |
| 230197009 | Acute viral transverse myelitis (disorder) | 47000000 | Acute transverse myelitis (disorder) | 1 | 1 |
| 701003 | Adult osteochondritis of spine (disorder) | 444578005 | Disorder of articular cartilage of vertebral column (disorder) | 1 | 1 |
| 293940009 | Allergy to prochlorperazine (finding) | 293928009 | Allergy to phenothiazine (finding) | 1 | 1 |

| | | | | | |
|---|---|---|---|---|---|
| 293941008 | Allergy to trifluoperazine (finding) | 293928009 | Allergy to phenothiazine (finding) | 1 | 1 |
| 311495006 | Amyloid disease of the urethra (disorder) | 236692005 | Degenerative disorder of urethra (disorder) | 1 | 1 |
| 2978006 | Aneurysm of conjunctiva (disorder) | 432119003 | Aneurysm (disorder) | 1 | 1 |
| 230707007 | Anterior cerebral circulation hemorrhagic infarction (disorder) | 230693009 | Anterior cerebral circulation infarction (disorder) | 1 | 1 |
| 225638007 | Anxiety about resuming sexual relations (finding) | 48079002 | Disturbance in mood (finding) | 1 | 1 |
| 405522001 | Aorto-femoral bifurcation graft for repair of aneurysm (procedure) | 233370007 | Aortic aneurysm repair (procedure) | 1 | 1 |
| 1074011000119100 | Arthritis of right great toe due to gout (disorder) | 428839004 | Arthritis of toe due to gout (disorder) | 1 | 1 |
| 719191003 | Atrophy of deltoid muscle (disorder) | 363056008 | Degenerative disorder of extremity (disorder) | 0.999999767 | 0.999965628 |
| 402819001 | Basal cell carcinoma of skin of lip (disorder) | 269515006 | Carcinoma of lip (disorder) | 0.999942128 | 0.96686637 |
| 723619005 | Behavioral couple psychotherapy (regime/therapy) | 166001 | Behavioral therapy (regime/therapy) | 1 | 1 |
| 92198006 | Benign neoplasm of lymph nodes of face (disorder) | 92098009 | Benign neoplasm of face (disorder) | 1 | 1 |

| | | | | | |
|---|---|---|---|---|---|
| 109938006 | Benign neoplasm of peripheral nerves of hip (disorder) | 92392000 | Benign neoplasm of soft tissues of hip (disorder) | 1 | 1 |
| 109938006 | Benign neoplasm of peripheral nerves of hip (disorder) | 109936005 | Benign neoplasm of peripheral nerves of lower limb (disorder) | 1 | 1 |
| 109944005 | Benign neoplasm of peripheral nerves of pelvis region (disorder) | 109946007 | Benign neoplasm of peripheral nerves of trunk (disorder) | 0.999997457 | 0.995867789 |
| 92112008 | Benign neoplasm of prepuce (disorder) | 92384009 | Benign neoplasm of skin (disorder) | 0.999999754 | 0.999999429 |
| 92336000 | Benign neoplasm of scrotum (disorder) | 92384009 | Benign neoplasm of skin (disorder) | 0.999970776 | 0.999919548 |
| 92362009 | Benign neoplasm of skin of forearm (disorder) | 448730001 | Benign neoplasm of soft tissue of forearm (disorder) | 0.999999529 | 0.999966194 |
| 724590008 | Benign osteogenic neoplasm of articular cartilage of clavicle (disorder) | 92061008 | Benign neoplasm of clavicle (disorder) | 1 | 1 |
| 254981002 | Benign tumor of acoustic vestibular nerve (disorder) | 387893006 | Neoplasm of acoustic vestibular nerve (disorder) | 0.999991535 | 0.99940831 |
| 49607006 | Biotin deficiency disease (disorder) | 413652008 | Biotin deficiency (disorder) | 0.999995725 | 0.999598598 |

| | | | | | |
|---|---|---|---|---|---|
| 84208005 | Blister of elbow without infection (disorder) | 44707000 | Superficial injury of elbow without infection (disorder) | 1 | 1 |
| 30888005 | Blister of gum with infection (disorder) | 76362004 | Superficial injury of gum with infection (disorder) | 0.99999913 | 0.982109467 |
| 38119007 | Blister of trunk without infection (disorder) | 28388008 | Superficial injury of trunk without infection (disorder) | 0.999984781 | 0.99751536 |
| 240301009 | Breastfeeding problem in the newborn (finding) | 78164000 | Feeding problem (finding) | 0.999118714 | 0.960118325 |
| 48689002 | Burn of male genitalia (disorder) | 284220000 | Burn of genitalia (disorder) | 0.999997737 | 0.994171576 |
| 128701000119108 | Capsulitis of tarsus (disorder) | 6858004 | Capsulitis (disorder) | 0.999999985 | 0.999998688 |
| 92540005 | Carcinoma in situ of areola of female breast (disorder) | 254838004 | Carcinoma of breast (disorder) | 1 | 1 |
| 92542002 | Carcinoma in situ of ascending colon (disorder) | 269533000 | Carcinoma of colon (disorder) | 0.999999986 | 0.999960905 |
| 271525004 | Carcinoma in situ of liver and/or biliary system (disorder) | 286887005 | Carcinoma liver and/or biliary system (disorder) | 0.999997139 | 0.99928121 |
| 92757006 | Carcinoma in situ of subglottis (disorder) | 372104008 | Carcinoma of subglottis (disorder) | 1 | 1 |
| 92791005 | Carcinoma in situ of vagina (disorder) | 254893005 | Carcinoma of vagina (disorder) | 0.99992935 | 0.956522888 |
| 254423005 | Carcinoma of lingual tonsil (disorder) | 93868009 | Primary malignant neoplasm of lingual tonsil (disorder) | 1 | 1 |

| | | | | | |
|---|---|---|---|---|---|
| 213215000 | Cardiorespiratory failure as a complication of care (disorder) | 233924009 | Heart failure as a complication of care (disorder) | 0.999999989 | 0.999998633 |
| 66731000119103 | Carrier of Duchenne muscular dystrophy (finding) | 137511000119103 | Carrier of muscular dystrophy (finding) | 0.999999915 | 0.975 |
| 52190008 | Cellulitis of scalp (disorder) | 128936008 | Bacterial infection of skin (disorder) | 0.999993195 | 0.999939461 |
| 204616008 | Central complete cleft palate with cleft lip (disorder) | 270513005 | Central complete cleft palate (disorder) | 1 | 1 |
| 365884000 | Cerebellar ataxic gait (finding) | 69021004 | Cerebellar gait (finding) | 0.99999988 | 0.999993059 |
| 230732009 | Cerebral arteritis in giant cell arteritis (disorder) | 230731002 | Cerebral arteritis in systemic vasculitis (disorder) | 0.999999912 | 0.99684432 |
| 231378009 | Chemical denervation of spinal facet joint of lumbar vertebra (procedure) | 448693009 | Denervation of spinal facet joint of lumbar vertebra (procedure) | 1 | 1 |
| 57940000 | Chronic duodenal ulcer without hemorrhage, without perforation AND without obstruction (disorder) | 38365000 | Peptic ulcer without hemorrhage, without perforation AND without obstruction (disorder) | 0.999993919 | 0.998974583 |
| 76796008 | Chronic gastric ulcer without hemorrhage AND without perforation (disorder) | 37442009 | Peptic ulcer without hemorrhage AND without perforation (disorder) | 0.999892679 | 0.985470972 |

| | | | | | |
|---|---|---|---|---|---|
| 122961000119105 | Chronic lymphoid leukemia in relapse (disorder) | 92812005 | Chronic leukemia, disease (disorder) | 0.999999951 | 0.999977693 |
| 1083371000119100 | Chronic nonsuppurative otitis media of bilateral ears (disorder) | 1083391000119100 | Chronic otitis media of bilateral ears (disorder) | 1 | 1 |
| 1091111000119100 | Chronic nonsuppurative otitis media of right ear (disorder) | 1091131000119100 | Chronic otitis media of right ear (disorder) | 1 | 1 |
| 1077611000119100 | Chronic osteomyelitis of right ankle (disorder) | 40970001 | Chronic osteomyelitis (disorder) | 1 | 1 |
| 193184006 | Chronic painful neuropathy due to diabetes mellitus (disorder) | 373621006 | Chronic pain syndrome (disorder) | 0.999997745 | 0.99993736 |
| 197763008 | Chronic pyonephrosis (disorder) | 63302006 | Chronic pyelonephritis (disorder) | 1 | 1 |
| 403328001 | Chronic telogen effluvium (disorder) | 128236002 | Chronic disease of skin (disorder) | 0.999999728 | 0.999994923 |
| 1075021000119100 | Chronic tophaceous gout of left foot (disorder) | 16076171000119100 | Tophus of left foot co-occurrent and due to gout (disorder) | 1 | 1 |
| 14248008 | Chronic vaginitis (disorder) | 128294001 | Chronic inflammatory disorder (disorder) | 0.999447666 | 0.998424581 |
| 14248008 | Chronic vaginitis (disorder) | 128290005 | Chronic disease of genitourinary system (disorder) | 0.999639082 | 0.997863811 |

| | | | | | |
|---|---|---|---|---|---|
| 117898009 | Circulating immune complexes conglutinin solid phase induced immune complex assay (procedure) | 252325003 | Circulating immune complexes assay (procedure) | 1 | 1 |
| 416176009 | Closed fracture dislocation of distal interphalangeal joint of finger (disorder) | 125802004 | Closed traumatic dislocation of interphalangeal joint of finger (disorder) | 0.999999385 | 0.999701071 |
| 704020004 | Closed fracture of sesamoid bone of foot (disorder) | 342070009 | Closed fracture of foot (disorder) | 0.99999993 | 0.999999176 |
| 209285008 | Closed fracture subluxation radiocarpal joint (disorder) | 429630006 | Closed dislocation of radiocarpal joint (disorder) | 1 | 1 |
| 209285008 | Closed fracture subluxation radiocarpal joint (disorder) | 209264008 | Closed fracture dislocation of wrist (disorder) | 1 | 1 |
| 125802004 | Closed traumatic dislocation of interphalangeal joint of finger (disorder) | 417349008 | Closed fracture dislocation of proximal interphalangeal joint of digit of hand (disorder) | 0.999698008 | 0.912144742 |
| 228557008 | Cognitive and behavioral therapy (regime/therapy) | 228553007 | Cognitive therapy (regime/therapy) | 1 | 1 |
| 716635007 | Cognitive communication disorder (disorder) | 443265004 | Cognitive disorder (disorder) | 0.999673091 | 0.977944363 |
| 84138006 | Collapse of vertebra (disorder) | 430886005 | Disorder of vertebra (disorder) | 0.99966515 | 0.987115347 |

| | | | | | |
|---|---|---|---|---|---|
| 104368007 | Complement decay accelerating factor measurement (procedure) | 104336004 | Complement component, antigen measurement (procedure) | 1 | 1 |
| 104347006 | Complement membrane C3b-C4b cofactor protein measurement (procedure) | 104336004 | Complement component, antigen measurement (procedure) | 1 | 1 |
| 36655007 | Complete ankylosis of the spine (disorder) | 430886005 | Disorder of vertebra (disorder) | 1 | 1 |
| 76842000 | Complete aphalangia of upper limb (disorder) | 48301005 | Congenital absence of finger (disorder) | 1 | 1 |
| 715853009 | Computed tomography of salivary gland with contrast (procedure) | 241532000 | Computed tomography sialogram (procedure) | 1 | 1 |
| 15932221000119100 | Congenital absence of right ovary (disorder) | 12017008 | Congenital absence of ovary (disorder) | 1 | 1 |
| 109521001 | Congenital alveolar hyperplasia of maxilla (disorder) | 28070007 | Congenital maxillary hyperplasia (disorder) | 0.999999949 | 0.999749078 |
| 33534005 | Congenital bowing of femur (disorder) | 716098006 | Congenital bowing of long bone (disorder) | 0.999995237 | 0.951093201 |
| 15670801000119100 | Congenital dislocation of left knee (disorder) | 59068006 | Congenital dislocation of knee (disorder) | 1 | 1 |
| 15670641000119100 | Congenital dislocation of right patella (disorder) | 205067002 | Congenital dislocation of patella (disorder) | 1 | 1 |

| | | | | | |
|---|---|---|---|---|---|
| 206529002 | Congenital fecaliths causing obstruction (disorder) | 3886001 | Congenital fecaliths (disorder) | 1 | 1 |
| 38827001 | Congenital fusion of spine (disorder) | 430886005 | Disorder of vertebra (disorder) | 0.999927462 | 0.997128804 |
| 287087003 | Congenital lordosis/scoliosis (disorder) | 205043005 | Congenital deformity of spine (disorder) | 0.999999615 | 0.999599868 |
| 234396004 | Congenital methemoglobinemia with abnormal methemoglobins (disorder) | 74912001 | Hereditary methemoglobinemia due to globin chain mutation (disorder) | 1 | 1 |
| 398943008 | Congenital pigmented melanocytic nevus of skin (disorder) | 400083002 | Congenital hamartoma of skin (disorder) | 0.999962886 | 0.941923069 |
| 342821000119103 | Congenital posterior subcapsular polar cataract (disorder) | 76562003 | Congenital subcapsular cataract (disorder) | 1 | 1 |
| 203070001 | Contracture of iliopsoas (disorder) | 280133005 | Disorder of soft tissue of trunk (disorder) | 0.999999858 | 0.999999634 |
| 23287003 | Contusion of scrotum (disorder) | 262526004 | Wound of skin (disorder) | 0.999999215 | 0.999996299 |
| 16350000 | Corneal macula interfering with central vision (disorder) | 95677002 | Disorder of vision (disorder) | 1 | 1 |
| 11853731000119100 | Crush injury of right ankle (disorder) | 23697004 | Crush syndrome (disorder) | 1 | 1 |
| 428870007 | Cystic adventitial disease of popliteal artery (disorder) | 363056008 | Degenerative disorder of extremity (disorder) | 0.999999942 | 0.999991406 |

| | | | | | |
|---|---|---|---|---|---|
| 27597005 | Deep third degree burn of female genitalia (disorder) | 447106005 | Deep third degree burn of genitalia (disorder) | 1 | 1 |
| 124149005 | Deficiency of 2-hydroxyglutarate dehydrogenase (disorder) | 124226002 | Deficiency of dehydrogenase (disorder) | 1 | 1 |
| 124534009 | Deficiency of adenosine triphosphate pyrophosphatase (disorder) | 124407008 | Deficiency of hydrolase (disorder) | 1 | 1 |
| 124481007 | Deficiency of adenosylhomocysteinase (disorder) | 124407008 | Deficiency of hydrolase (disorder) | 1 | 1 |
| 124363008 | Deficiency of alcohol sulfotransferase (disorder) | 124402002 | Deficiency of sulfotransferase (disorder) | 1 | 1 |
| 124565000 | Deficiency of alpha-aminoacylpeptide hydrolase (disorder) | 124407008 | Deficiency of hydrolase (disorder) | 1 | 1 |
| 124600004 | Deficiency of aromatic-L-amino-acid decarboxylase (disorder) | 124592006 | Deficiency of lyase (disorder) | 1 | 1 |
| 124465002 | Deficiency of beta-galactosidase (disorder) | 124407008 | Deficiency of hydrolase (disorder) | 1 | 1 |
| 124157008 | Deficiency of betaine-aldehyde dehydrogenase (disorder) | 124226002 | Deficiency of dehydrogenase (disorder) | 1 | 1 |
| 124678007 | Deficiency of bisphosphoglycerate mutase (disorder) | 124657009 | Deficiency of isomerase (disorder) | 1 | 1 |
| 124493003 | Deficiency of carboxypeptidase B (disorder) | 124563007 | Deficiency of peptide hydrolase (disorder) | 1 | 1 |

| | | | | | |
|---|---|---|---|---|---|
| 124703008 | Deficiency of cholate-CoA ligase (disorder) | 124687003 | Deficiency of ligase (disorder) | 1 | 1 |
| 124349002 | Deficiency of deoxyribonucleic acid-directed ribonucleic acid polymerase (disorder) | 124406004 | Deficiency of nucleotidyltransferase (disorder) | 1 | 1 |
| 124254009 | Deficiency of glucosamine-phosphate acetyltransferase (disorder) | 124382004 | Deficiency of transacylase (disorder) | 1 | 1 |
| 124143006 | Deficiency of glucose oxidase (disorder) | 124228001 | Deficiency of oxidase (disorder) | 1 | 1 |
| 124596009 | Deficiency of glutamate decarboxylase (disorder) | 124592006 | Deficiency of lyase (disorder) | 1 | 1 |
| 124264000 | Deficiency of glycerol-3-phosphate acyltransferase (disorder) | 124382004 | Deficiency of transacylase (disorder) | 1 | 1 |
| 302661005 | Deficiency of glycosidase (disorder) | 124407008 | Deficiency of hydrolase (disorder) | 1 | 1 |
| 124626009 | Deficiency of heparin lyase (disorder) | 124592006 | Deficiency of lyase (disorder) | 1 | 1 |
| 124605009 | Deficiency of ketotetrose-phosphate aldolase (disorder) | 124592006 | Deficiency of lyase (disorder) | 1 | 1 |
| 124659007 | Deficiency of lactate racemase (disorder) | 124657009 | Deficiency of isomerase (disorder) | 1 | 1 |
| 124124006 | Deficiency of malate dehydrogenase (disorder) | 124226002 | Deficiency of dehydrogenase (disorder) | 1 | 1 |
| 124142001 | Deficiency of malate oxidase (disorder) | 124228001 | Deficiency of oxidase (disorder) | 1 | 1 |

| | | | | | |
|---|---|---|---|---|---|
| 124365001 | Deficiency of malonate CoA-transferase (disorder) | 124404001 | Deficiency of coenzyme-A transferase (disorder) | 1 | 1 |
| 124266003 | Deficiency of maltose phosphorylase (disorder) | 124388000 | Deficiency of hexosyltransferase (disorder) | 1 | 1 |
| 124680001 | Deficiency of methylmalonyl-coenzyme A mutase (disorder) | 124657009 | Deficiency of isomerase (disorder) | 1 | 1 |
| 124296008 | Deficiency of oximinotransferase (disorder) | 124237001 | Deficiency of transferase (disorder) | 1 | 1 |
| 124347000 | Deficiency of pantetheine-phosphate adenylyltransferase (disorder) | 124406004 | Deficiency of nucleotidyltransferase (disorder) | 1 | 1 |
| 124505003 | Deficiency of peptidoglycan endopeptidase (disorder) | 124407008 | Deficiency of hydrolase (disorder) | 1 | 1 |
| 124676006 | Deficiency of phosphoglucomutase (disorder) | 124657009 | Deficiency of isomerase (disorder) | 1 | 1 |
| 124712005 | Deficiency of phosphoribosylamine-glycine ligase (disorder) | 124687003 | Deficiency of ligase (disorder) | 1 | 1 |
| 124442007 | Deficiency of phosphorylase phosphatase (disorder) | 124407008 | Deficiency of hydrolase (disorder) | 1 | 1 |
| 361010007 | Deficiency of proline dipeptidase (disorder) | 124563007 | Deficiency of peptide hydrolase (disorder) | 1 | 1 |
| 124593001 | Deficiency of pyruvate decarboxylase (disorder) | 124592006 | Deficiency of lyase (disorder) | 1 | 1 |
| 124573009 | Deficiency of ribonuclease (disorder) | 124407008 | Deficiency of hydrolase (disorder) | 1 | 1 |

| | | | | | |
|---|---|---|---|---|---|
| 124697007 | Deficiency of serine-transfer ribonucleic acid ligase (disorder) | 124687003 | Deficiency of ligase (disorder) | 1 | 1 |
| 124496006 | Deficiency of thrombin (disorder) | 124563007 | Deficiency of peptide hydrolase (disorder) | 1 | 1 |
| 124689000 | Deficiency of tryptophan-transfer ribonucleic acid ligase (disorder) | 124687003 | Deficiency of ligase (disorder) | 1 | 1 |
| 124153007 | Deficiency of uracil dehydrogenase (disorder) | 124226002 | Deficiency of dehydrogenase (disorder) | 1 | 1 |
| 124276000 | Deficiency of uracil phosphoribosyltransferase (disorder) | 124390004 | Deficiency of pentosyltransferase (disorder) | 1 | 1 |
| 124147007 | Deficiency of xanthine oxidase (disorder) | 124228001 | Deficiency of oxidase (disorder) | 1 | 1 |
| 16045391000119100 | Derangement of left wrist joint (disorder) | 146231000119109 | Derangement of joint of hand (disorder) | 1 | 1 |
| 394684000 | Diabetic pre-pregnancy education (procedure) | 6143009 | Diabetic education (procedure) | 1 | 1 |
| 310825003 | Difficulty inferring meaning (finding) | 288608007 | Difficulty using the elements of language (finding) | 1 | 1 |
| 286499006 | Difficulty preparing feed (finding) | 286493007 | Difficulty preparing meal (finding) | 1 | 1 |

| | | | | | |
|---|---|---|---|---|---|
| 716486007 | Difficulty using consonant vowel consonant vowel combination (finding) | 716539005 | Finding related to ability to use consonant vowel consonant vowel combination (finding) | 1 | 1 |
| 403496009 | Discoid lupus erythematosus of genital mucous membranes (disorder) | 403494007 | Discoid lupus erythematosus of mucous membranes (disorder) | 1 | 1 |
| 402992008 | Disseminated actinomycosis infection affecting skin (disorder) | 24779007 | Disseminated actinomycosis (disorder) | 1 | 1 |
| 304108004 | Distant osseomyocutaneous flap (procedure) | 265659001 | Distant myocutaneous flap (procedure) | 1 | 1 |
| 253568009 | Doubly committed subarterial ventricular septal defect (disorder) | 448876006 | Subarterial ventricular septal defect (disorder) | 0.999999956 | 0.999996834 |
| 672531000119106 | Dysphasia due to and following hemorrhagic cerebrovascular accident (disorder) | 16260551000119100 | Dysphasia due to and following cerebrovascular accident (disorder) | 1 | 1 |
| 300138002 | Ear canal problem (finding) | 300197009 | Ear problem (finding) | 0.999999166 | 0.999894887 |
| 427581001 | Electrocardiogram: ectopic beats with multifocal premature ventricular contractions (finding) | 427172004 | Electrocardiogram: premature ventricular contractions (finding) | 1 | 1 |

| | | | | | |
|---|---|---|---|---|---|
| 175690001 | Endarterectomy of popliteal artery and patch repair of popliteal artery (procedure) | 310618007 | Patch repair of popliteal artery (procedure) | 1 | 1 |
| 177052007 | Endoscopic biopsy of lesion of ovary (procedure) | 708747005 | Laparoscopic biopsy of ovary (procedure) | 1 | 1 |
| 710727006 | Enlargement of root of tongue (disorder) | 25273001 | Enlargement of tongue (disorder) | 1 | 1 |
| 254635004 | Epithelioid hemangioendothelioma of lung (disorder) | 254625005 | Malignant tumor of lung parenchyma (disorder) | 1 | 1 |
| 15632771000119100 | Exotropia of left eye (disorder) | 456171000124102 | Disorder of left eye (disorder) | 0.999999824 | 0.999994521 |
| 11454006 | Failed attempted abortion with amniotic fluid embolism (disorder) | 198812002 | Failed attempted abortion with embolism (disorder) | 1 | 1 |
| 33490001 | Failed attempted abortion with fat embolism (disorder) | 198812002 | Failed attempted abortion with embolism (disorder) | 1 | 1 |
| 42021008 | Familial diabetes insipidus (disorder) | 111941005 | Familial disease (disorder) | 0.999999867 | 0.999633431 |
| 236460004 | Familial proximal renal tubular acidosis (disorder) | 111941005 | Familial disease (disorder) | 1 | 1 |
| 402335001 | Familial psoriasis (disorder) | 111941005 | Familial disease (disorder) | 0.999999912 | 0.999755501 |
| 289459009 | Finding of arrangement of female pelvis (finding) | 299659002 | Finding of arrangement of pelvis (finding) | 1 | 1 |

| | | | | | |
|---|---|---|---|---|---|
| 300475002 | Finding of measures of urine output (finding) | 300474003 | Finding of urine output (finding) | 0.999999599 | 0.999997242 |
| 364984007 | Finding related to ability to release grip (finding) | 364976008 | Finding related to ability to grip (finding) | 1 | 1 |
| 231883009 | Foreign body in posterior wall eye (disorder) | 450671000124109 | Foreign body in eye (disorder) | 0.999999684 | 0.99996408 |
| 263065002 | Fracture dislocation of costovertebral joint (disorder) | 263063009 | Fracture dislocation of joint (disorder) | 1 | 1 |
| 127283002 | Fracture of ethmoid bone (disorder) | 125593007 | Injury of face (disorder) | 0.999999946 | 0.999999697 |
| 263099004 | Fracture subluxation of acromioclavicular joint (disorder) | 281519006 | Fracture subluxation of joint of upper limb (disorder) | 1 | 1 |
| 20659000 | Full thickness burn of female genitalia (disorder) | 211792006 | Full thickness burn of genitalia (disorder) | 0.999999615 | 0.969251318 |
| 66554005 | Full thickness burn of male genitalia (disorder) | 211792006 | Full thickness burn of genitalia (disorder) | 0.999996691 | 0.956945312 |
| 77490007 | Full thickness burn of shoulder (disorder) | 403192003 | Full thickness burn (disorder) | 1 | 1 |
| 308661000119100 | Ganglion cyst of left wrist (disorder) | 202942009 | Ganglion of wrist (disorder) | 0.999999973 | 0.999999346 |
| 73481001 | Gastric ulcer without hemorrhage AND without perforation (disorder) | 37442009 | Peptic ulcer without hemorrhage AND without perforation (disorder) | 0.998784341 | 0.949358738 |
| 708964003 | Gastritis cystica profunda (disorder) | 4556007 | Gastritis (disorder) | 1 | 1 |

| | | | | | |
|---|---|---|---|---|---|
| 283237004 | Glass in lip (disorder) | 283236008 | Glass in mouth (disorder) | 1 | 1 |
| 29538005 | Glaucomatocyclitic crisis (disorder) | 21928008 | Secondary open-angle glaucoma (disorder) | 0.999998433 | 0.99821669 |
| 403971002 | Glomangiomyoma of skin (disorder) | 254795008 | Glomus tumor of skin (disorder) | 1 | 1 |
| 90871000 | Grafting of fascia to tarsal cartilage (procedure) | 68012008 | Grafting of fascia of eyelid (procedure) | 1 | 1 |
| 180758009 | Harvest of flap of skin and muscle of trunk (procedure) | 180793002 | Harvest of flap of muscle of trunk (procedure) | 1 | 1 |
| 300223008 | Hearing for conversational voice impaired (finding) | 300227009 | Hearing for voice impaired (finding) | 0.999999934 | 0.999993985 |
| 429176009 | Hemangioendothelioma of abdomen (disorder) | 126757004 | Neoplasm of blood vessel of abdomen (disorder) | 0.999999798 | 0.977753313 |
| 254923001 | Hemangiopericytoma of kidney (disorder) | 134335004 | Hemangiopericytoma (disorder) | 1 | 1 |
| 277522009 | Hemangiopericytoma of meninges (disorder) | 134335004 | Hemangiopericytoma (disorder) | 1 | 1 |
| 31925001 | Hereditary factor I deficiency disease (disorder) | 439458000 | Factor I deficiency disease (disorder) | 1 | 1 |
| 111859007 | Herpes zoster without complication (disorder) | 4740000 | Herpes zoster (disorder) | 0.999999639 | 0.999995317 |
| 250952007 | Hyperkinetic right ventricular wall (finding) | 371855002 | Hyperkinetic ventricular wall (finding) | 1 | 1 |

| | | | | | |
|---|---|---|---|---|---|
| 22966008 | Hypertensive heart AND renal disease complicating AND/OR reason for care during pregnancy (disorder) | 20753005 | Hypertensive heart disease complicating AND/OR reason for care during pregnancy (disorder) | 1 | 1 |
| 348241000119108 | Hypertropia of right eye (disorder) | 456161000124109 | Disorder of right eye (disorder) | 0.999998397 | 0.999953938 |
| 721793008 | Infection caused by Saprochaete capitata (disorder) | 721803008 | Infection caused by Saprochaete (disorder) | 1 | 1 |
| 203015004 | Infective myositis of pelvis (disorder) | 609620004 | Disorder of pelvis (disorder) | 1 | 1 |
| 254840009 | Inflammatory carcinoma of breast (disorder) | 266579006 | Inflammatory disorder of breast (disorder) | 0.999999308 | 0.999966559 |
| 195929004 | Influenza with gastrointestinal tract involvement (disorder) | 6142004 | Influenza (disorder) | 1 | 1 |
| 68242000 | Injection of sympathetic ciliary ganglion (procedure) | 89575006 | Injection of sympathetic ganglion (procedure) | 1 | 1 |
| 609593002 | Injury of branch of median nerve (disorder) | 67279004 | Injury of median nerve (disorder) | 1 | 1 |
| 212307006 | Injury of cutaneous sensory nerve at forearm level (disorder) | 721531006 | Injury of nerve at forearm level (disorder) | 0.999996092 | 0.998759584 |
| 262715008 | Injury of spinal cord vasculature (disorder) | 90584004 | Spinal cord injury (disorder) | 1 | 1 |

| | | | | | |
|---|---|---|---|---|---|
| 726227002 | Injury of tendon of adductor muscle of thigh (disorder) | 95847005 | Injury of muscle (disorder) | 1 | 1 |
| 307731004 | Injury of tendon of the rotator cuff of shoulder (disorder) | 444003007 | Disorder of joint of shoulder region (disorder) | 0.999980431 | 0.999858995 |
| 262937009 | Injury to blood vessel of neck (disorder) | 280132000 | Disorder of soft tissue of neck (disorder) | 0.999968137 | 0.999570821 |
| 236083006 | Intermittent vomiting (disorder) | 63722008 | Chronic vomiting (disorder) | 0.999998446 | 0.999804503 |
| 292891000119104 | Intracranial granuloma (disorder) | 39367000 | Inflammatory disease of the central nervous system (disorder) | 0.999999982 | 0.999998729 |
| 290441000119105 | Intractable chronic headache following trauma (disorder) | 290431000119101 | Intractable headache following trauma (disorder) | 1 | 1 |
| 235839006 | Irritable bowel syndrome variant of childhood with diarrhea (disorder) | 197125005 | Irritable bowel syndrome with diarrhea (disorder) | 1 | 1 |
| 193436005 | Juxtapapillary focal chorioretinitis (disorder) | 699516005 | Juxtapapillary focal retinitis (disorder) | 0.999999814 | 0.999982962 |
| 35625006 | Keratosis of middle ear (disorder) | 254666005 | Keratosis (disorder) | 1 | 1 |
| 724790005 | Laceration of fascia of neck (disorder) | 283364000 | Laceration of neck (disorder) | 1 | 1 |
| 10963531000119100 | Laceration of finger of right hand (disorder) | 10956681000119100 | Open wound of finger of right hand (disorder) | 1 | 1 |

| | | | | | |
|---|---|---|---|---|---|
| 724979009 | Laceration of tendon of extensor muscle of finger at forearm level (disorder) | 724973005 | Injury of tendon of extensor muscle of finger at forearm level (disorder) | 0.999999958 | 0.999995989 |
| 402947001 | Late secondary syphilis (disorder) | 72083004 | Late syphilis (disorder) | 1 | 1 |
| 88477005 | Lateral developmental cyst of jaw (disorder) | 73573004 | Congenital anomaly of musculoskeletal system (disorder) | 1 | 1 |
| 6403005 | Lengthening of muscle of hand (procedure) | 9150007 | Change of length of muscle of hand (procedure) | 1 | 1 |
| 298149009 | Lesion of joint (finding) | 300577008 | Finding of lesion (finding) | 0.904621437 | 0.667116139 |
| 238655003 | Lichen planus of scalp (disorder) | 402694007 | Dermatosis of scalp (disorder) | 0.999999583 | 0.987880886 |
| 23869008 | Literal paraphasia (finding) | 53096005 | Paraphasia (finding) | 1 | 1 |
| 52837007 | Longitudinal deficiency of femur (disorder) | 271092002 | Deformity of femur (disorder) | 0.999997685 | 0.996256144 |
| 313371000119106 | Loose body in joint of left shoulder region (disorder) | 202156005 | Loose body in shoulder joint (disorder) | 1 | 1 |
| 313441000119106 | Loose body in joint of right shoulder region (disorder) | 202156005 | Loose body in shoulder joint (disorder) | 1 | 1 |
| 67437007 | Lumbar spondylosis with myelopathy (disorder) | 239880009 | Lumbar spondylosis (disorder) | 0.999617134 | 0.99624857 |
| 447805007 | Lymphoma of greater curvature of stomach (disorder) | 448555009 | Lymphoma of body of stomach (disorder) | 1 | 1 |

| | | | | | |
|---|---|---|---|---|---|
| 431627008 | Magnetic resonance imaging of small intestine (procedure) | 241621009 | Magnetic resonance imaging of abdomen (procedure) | 1 | 1 |
| 93648001 | Malignant melanoma of skin of temporal region (disorder) | 93646002 | Malignant melanoma of skin of scalp (disorder) | 0.999999959 | 0.999948531 |
| 188268004 | Malignant neoplasm of connective tissue of orbit (disorder) | 448274000 | Malignant neoplasm of connective tissue (disorder) | 1 | 1 |
| 363470000 | Malignant neoplasm of occipital lobe (disorder) | 188282004 | Malignant neoplasm of cerebral cortex (disorder) | 0.999991026 | 0.973265288 |
| 254980001 | Malignant tumor of acoustic vestibular nerve (disorder) | 387893006 | Neoplasm of acoustic vestibular nerve (disorder) | 0.999999569 | 0.99996921 |
| 363377003 | Malignant tumor of lingual tonsil (disorder) | 363393007 | Malignant tumor of tonsil (disorder) | 0.999994772 | 0.998532359 |
| 109685003 | Maxillary alveolar hypoplasia (disorder) | 15492000 | Atrophy of edentulous maxillary alveolar ridge (disorder) | 0.999996584 | 0.997948892 |
| 292230006 | Mercaptopurine adverse reaction (disorder) | 292255000 | Immunosuppressant adverse reaction (disorder) | 0.999999958 | 0.998039216 |
| 133851000119107 | Merkel cell carcinoma of neck (disorder) | 94058008 | Primary malignant neoplasm of soft tissues of neck (disorder) | 1 | 1 |
| 720451004 | Minimal recurrent major depression (disorder) | 718636001 | Minimal depression (disorder) | 0.999999897 | 0.99998711 |

| | | | | | |
|---|---|---|---|---|---|
| 61568004 | Miscarriage with salpingo-oophoritis (disorder) | 7910003 | Miscarriage with salpingitis (disorder) | 1 | 1 |
| 720452006 | Moderately severe recurrent major depression (disorder) | 719592004 | Moderately severe major depression (disorder) | 0.999995649 | 0.930729167 |
| 403950007 | MollÂ' s gland adenocarcinoma (disorder) | 93663003 | Primary malignant neoplasm of skin with adnexal differentiation (disorder) | 1 | 1 |
| 723408004 | Multifocal pattern dystrophy of retinal pigment epithelium simulating fundus flavimaculatus (disorder) | 41799005 | Hereditary retinal dystrophy (disorder) | 1 | 1 |
| 735871006 | Multiple open wounds of ankle (disorder) | 735900007 | Multiple injuries of ankle (disorder) | 0.999999933 | 0.988094085 |
| 89801006 | Multiple traumatic closed dislocations of upper arm (disorder) | 125595000 | Injury of upper arm (disorder) | 1 | 1 |
| 126990005 | Neoplasm of peripheral nerves of hip (disorder) | 126989001 | Neoplasm of peripheral nerves of lower limb (disorder) | 1 | 1 |
| 95003003 | Neoplasm of uncertain behavior of pisiform bone of hand (disorder) | 94861008 | Neoplasm of uncertain behavior of hand (disorder) | 1 | 1 |

| | | | | | |
|---|---|---|---|---|---|
| 95003003 | Neoplasm of uncertain behavior of pisiform bone of hand (disorder) | 126654000 | Neoplasm of hand (disorder) | 1 | 1 |
| 713457002 | Neovascular glaucoma due to diabetes mellitus (disorder) | 860798008 | Glaucoma due to diabetes mellitus (disorder) | 0.999999968 | 0.98 |
| 17262008 | Non-alcoholic Korsakoff's psychosis (disorder) | 69482004 | Korsakoff's psychosis (disorder) | 0.999998146 | 0.989963694 |
| 299157001 | Normal passive range of thumb extension (finding) | 123621006 | Normal extension (finding) | 1 | 1 |
| 449127009 | Normal separation of commissures of aortic valve (finding) | 301100007 | Aortic valve normal (finding) | 1 | 1 |
| 102967008 | Normal visual evoked potential (finding) | 102961009 | Normal evoked potential (finding) | 1 | 1 |
| 247877002 | Obsessional thoughts of poisoning (finding) | 247880001 | Obsessional thoughts of disease (finding) | 1 | 1 |
| 454031000124106 | Occlusion of superior cerebellar artery (disorder) | 2929001 | Occlusion of artery (disorder) | 1 | 1 |
| 208414008 | Open fracture finger metacarpal base (disorder) | 208418006 | Open fracture finger metacarpal (disorder) | 0.999997858 | 0.997162844 |
| 10836901000119100 | Open fracture of multiple left ribs (disorder) | 28081005 | Open fracture of multiple ribs (disorder) | 1 | 1 |
| 32988004 | Open fracture of multiple sites of phalanges of hand (disorder) | 21698002 | Open fracture of phalanx of finger (disorder) | 0.999998166 | 0.999923251 |

| | | | | | |
|---|---|---|---|---|---|
| 10823131000119100 | Open fracture of proximal phalanx of lesser toe of left foot (disorder) | 10823211000119100 | Open fracture of phalanx of lesser toe of left foot (disorder) | 1 | 1 |
| 10815901000119100 | Open fracture of proximal phalanx of lesser toe of right foot (disorder) | 10815981000119100 | Open fracture of phalanx of lesser toe of right foot (disorder) | 1 | 1 |
| 209380001 | Open fracture subluxation of subtalar joint (disorder) | 209381002 | Open fracture subluxation of midtarsal joint (disorder) | 1 | 1 |
| 10959641000119100 | Open wound of left elbow due to human bite (disorder) | 125643001 | Open wound (disorder) | 1 | 1 |
| 10957361000119100 | Open wound of left thumb due to human bite (disorder) | 125643001 | Open wound (disorder) | 1 | 1 |
| 10953481000119100 | Open wound of right little finger due to cat bite (disorder) | 10953921000119100 | Open wound of right hand due to cat bite (disorder) | 1 | 1 |
| 10964031000119100 | Open wound of right upper arm due to bite (disorder) | 733402009 | Open bite of upper arm (disorder) | 1 | 1 |
| 234998009 | Orofacial granulomatosis of gingivae (disorder) | 235048000 | Orofacial granulomatosis (disorder) | 1 | 1 |
| 323251000119103 | Osteoarthritis of first carpometacarpal joint of left hand (disorder) | 1074801000119100 | Arthritis of first carpometacarpal joint of left hand (disorder) | 0.999992938 | 0.953488285 |
| 239873007 | Osteoarthritis of knee (disorder) | 371081002 | Arthritis of knee (disorder) | 0.966800121 | 0.8732817 |

| | | | | | |
|---|---|---|---|---|---|
| 1074451000119100 | Osteoarthritis of left sternoclavicular joint (disorder) | 1074661000119100 | Arthritis of left sternoclavicular joint (disorder) | 1 | 1 |
| 15722441000119100 | Osteoarthritis of left subtalar joint (disorder) | 16015791000119100 | Arthritis of left subtalar joint (disorder) | 1 | 1 |
| 82562007 | Osteochondritis dissecans (disorder) | 70736000 | Osteochondritis (disorder) | 0.999465535 | 0.986805124 |
| 78960005 | Pancreatic triacylglycerol lipase deficiency (disorder) | 302920007 | Pancreatic malabsorption (disorder) | 0.999999914 | 0.999762119 |
| 80513001 | Partial laryngectomy (procedure) | 38829003 | Partial excision (procedure) | 0.999999611 | 0.956521739 |
| 384680006 | Patch enlargement of coronary artery (procedure) | 384681005 | Patch repair of coronary artery (procedure) | 1 | 1 |
| 704335006 | Pathological fracture of humerus due to osteoporosis (disorder) | 268029009 | Pathological fracture (disorder) | 0.999991142 | 0.999044183 |
| 704338008 | Pathological fracture of tibia due to osteoporosis (disorder) | 445526000 | Pathological fracture of tibia (disorder) | 0.999999975 | 0.999941003 |
| 231851003 | Penetrating wound of orbit proper (disorder) | 315296002 | Penetrating wound of eye (disorder) | 0.999887036 | 0.981595775 |
| 233273007 | Percutaneous balloon angioplasty of superior mesenteric artery (procedure) | 447743004 | Percutaneous transluminal angioplasty of superior mesenteric artery (procedure) | 1 | 1 |

| | | | | | |
|---|---|---|---|---|---|
| 431659001 | Percutaneous transluminal angioplasty of common carotid artery using fluoroscopic guidance with contrast (procedure) | 175380003 | Percutaneous transluminal angioplasty of carotid artery (procedure) | 1 | 1 |
| 431660006 | Percutaneous transluminal angioplasty of common femoral artery using fluoroscopic guidance with contrast (procedure) | 175715003 | Percutaneous transluminal angioplasty of femoral artery (procedure) | 1 | 1 |
| 713155002 | Percutaneous transluminal balloon angioplasty with insertion of stent into carotid artery (procedure) | 425611003 | Percutaneous transluminal insertion of stent into carotid artery (procedure) | 1 | 1 |
| 712817003 | Percutaneous transluminal cutting balloon angioplasty of pulmonary vein (procedure) | 276892002 | Percutaneous balloon angioplasty of pulmonary vein (procedure) | 1 | 1 |
| 473367000 | Pericardial cyst along right cardiophrenic angle (disorder) | 609302002 | Pericardial cyst (disorder) | 0.999999519 | 0.996022356 |
| 46676000 | Pericarditis co-occurrent and due to benign primary tumor (disorder) | 16876006 | Pericarditis co-occurrent and due to primary tumor (disorder) | 1 | 1 |
| 55879006 | Pericarditis co-occurrent and due to malignant primary tumor (disorder) | 16876006 | Pericarditis co-occurrent and due to primary tumor (disorder) | 1 | 1 |

| | | | | | |
|---|---|---|---|---|---|
| 201182003 | Perifolliculitis capitis abscedens (disorder) | 83341004 | Perifolliculitis (disorder) | 0.999994849 | 0.949290061 |
| 403823001 | Periungual Ã¯Â¬Âbroma in tuberous sclerosis (disorder) | 280134004 | Disorder of soft tissue of limb (disorder) | 1 | 1 |
| 714251006 | Philadelphia chromosome-negative precursor B-cell acute lymphoblastic leukemia (disorder) | 277571004 | B-cell acute lymphoblastic leukemia (disorder) | 1 | 1 |
| 333141000119106 | Phlegmonous dacryocystitis of right eye (disorder) | 456161000124109 | Disorder of right eye (disorder) | 1 | 1 |
| 186792002 | Plasmodium vivax malaria with rupture of spleen (disorder) | 128070006 | Infectious disease of abdomen (disorder) | 0.999999854 | 0.999998696 |
| 35896003 | Plastic operation on hand with graft of muscle (procedure) | 71660004 | Repair of muscle of hand (procedure) | 1 | 1 |
| 243059005 | Poisoning due to nerve gas (disorder) | 75478009 | Poisoning (disorder) | 1 | 1 |
| 105151000119105 | Postoperative heterotopic calcification of skeletal muscle (disorder) | 450431000124108 | Postoperative heterotopic calcification of muscle (disorder) | 1 | 1 |
| 40511000119107 | Postpartum pre-existing essential hypertension (disorder) | 72022006 | Essential hypertension in obstetric context (disorder) | 0.999999942 | 0.99914966 |
| 40511000119107 | Postpartum pre-existing essential hypertension (disorder) | 72022006 | Essential hypertension in obstetric context (disorder) | 0.999999942 | 0.99914966 |

| | | | | | |
|---|---|---|---|---|---|
| 191454001 | Presenile dementia with paranoia (disorder) | 31081000119101 | Presenile dementia with delusions (disorder) | 0.99999997 | 0.99998494 |
| 225561003 | Pressure ulcer of heel (disorder) | 10635431000119100 | Chronic ulcer of heel (disorder) | 0.999243514 | 0.955561028 |
| 707485006 | Primary adenosquamous carcinoma of hypopharynx (disorder) | 707492001 | Primary squamous cell carcinoma of hypopharynx (disorder) | 1 | 1 |
| 707424007 | Primary adenosquamous cell carcinoma of larynx (disorder) | 276975007 | Carcinoma of larynx (disorder) | 1 | 1 |
| 93799003 | Primary malignant neoplasm of Ã¯Â¬Âbula (disorder) | 449628003 | Malignant neoplasm of long bone of lower leg (disorder) | 1 | 1 |
| 93742006 | Primary malignant neoplasm of cartilage of nose (disorder) | 94053004 | Primary malignant neoplasm of soft tissues of face (disorder) | 0.999999966 | 0.999999113 |
| 93807001 | Primary malignant neoplasm of frontal lobe (disorder) | 188282004 | Malignant neoplasm of cerebral cortex (disorder) | 0.999940776 | 0.968232105 |
| 93843007 | Primary malignant neoplasm of islets of Langerhans (disorder) | 187794005 | Malignant tumor of Islets of Langerhans (disorder) | 0.999995237 | 0.999658938 |
| 93868009 | Primary malignant neoplasm of lingual tonsil (disorder) | 372020000 | Primary malignant neoplasm of tonsil (disorder) | 0.999993275 | 0.998132618 |
| 93899005 | Primary malignant neoplasm of muscle of abdomen (disorder) | 148911000119107 | Primary malignant neoplasm of abdomen (disorder) | 1 | 1 |

| | | | | | |
|---|---|---|---|---|---|
| 109918005 | Primary malignant neoplasm of peripheral nerves and peripheral autonomic nervous system (disorder) | 128123007 | Disorder of peripheral autonomic nervous system (disorder) | 0.999999971 | 0.999871663 |
| 109933002 | Primary malignant neoplasm of peripheral nerves of shoulder (disorder) | 94005003 | Primary malignant neoplasm of shoulder (disorder) | 1 | 1 |
| 372009000 | Primary malignant neoplasm of scrotum (disorder) | 94047004 | Primary malignant neoplasm of skin (disorder) | 0.999988837 | 0.999916627 |
| 197441003 | Primary sclerosing cholangitis (disorder) | 17266006 | Primary cholangitis (disorder) | 0.999975668 | 0.997386967 |
| 707359008 | Primary squamous cell carcinoma of ethmoidal sinus (disorder) | 707346000 | Primary carcinoma of ethmoidal sinus (disorder) | 0.99999999 | 0.99999941 |
| 722673007 | Primary squamous cell carcinoma of lingual tonsil (disorder) | 254423005 | Carcinoma of lingual tonsil (disorder) | 0.999999962 | 0.99461889 |
| 724467001 | Primary squamous cell carcinoma of overlapping lesion of urinary organs (disorder) | 255066001 | Carcinoma of genitourinary organ (disorder) | 1 | 1 |
| 722674001 | Primary squamous cell carcinoma of parotid gland (disorder) | 254462001 | Carcinoma of parotid gland (disorder) | 0.999999899 | 0.996254834 |

| | | | | | |
|---|---|---|---|---|---|
| 175694005 | Profundoplasty of popliteal artery and patch repair of popliteal artery (procedure) | 310618007 | Patch repair of popliteal artery (procedure) | 1 | 1 |
| 230239001 | Progressive cerebellar ataxia with palatal myoclonus (disorder) | 230233000 | Progressive cerebellar ataxia (disorder) | 1 | 1 |
| 276550000 | Prolonged newborn physiological jaundice (disorder) | 276549000 | Newborn physiological jaundice (disorder) | 0.999999931 | 0.999997313 |
| 25297005 | Psychogenic adductor spastic dysphonia (disorder) | 275472008 | Psychogenic dysphonia (disorder) | 1 | 1 |
| 95929000 | Psychologically abused elder (finding) | 207871000119100 | Adult victim of emotional abuse (finding) | 1 | 1 |
| 25948008 | Pyloric antral stenosis (disorder) | 367403001 | Pyloric stenosis (disorder) | 1 | 1 |
| 678741000119108 | Quadrantanopia of right eye (finding) | 16059071000119100 | Visual field defect of right eye (finding) | 1 | 1 |
| 66769009 | Radiography of cervical spine (procedure) | 303938002 | Plain film of neck (procedure) | 1 | 1 |
| 252431001 | Radionuclide cardiac shunt study (procedure) | 85606007 | Radionuclide cardiac ventriculography (procedure) | 1 | 1 |
| 430266004 | Radionuclide two-phase bone imaging of wrist (procedure) | 430832002 | Radionuclide two-phase bone imaging of upper limb (procedure) | 1 | 1 |
| 236703003 | Recurrent infective cystitis (disorder) | 236622000 | Chronic infective cystitis (disorder) | 0.999998953 | 0.992062774 |

| | | | | | |
|---|---|---|---|---|---|
| 707332000 | Recurrent proliferative glomerulonephritis (disorder) | 195791000119101 | Chronic proliferative glomerulonephritis (disorder) | 0.999999607 | 0.999407977 |
| 429181000124108 | Refractory acute confusional migraine (disorder) | 423894005 | Refractory migraine (disorder) | 1 | 1 |
| 22073001 | Regular wired jaw diet (finding) | 42289002 | Wired jaw diet (finding) | 1 | 1 |
| 363726008 | Removal of foreign body of cornea by incision (procedure) | 62935003 | Incision of cornea (procedure) | 1 | 1 |
| 450583003 | Removal of slough from skin of neck (procedure) | 697948006 | Toilet of skin of neck (procedure) | 1 | 1 |
| 102830001 | Renal angle tenderness (finding) | 274279008 | Renal pain (finding) | 0.999985906 | 0.999489699 |
| 102830001 | Renal angle tenderness (finding) | 102831002 | Renal angle pain (finding) | 0.999980452 | 0.999145101 |
| 198949009 | Renal hypertension complicating pregnancy, childbirth and the puerperium (disorder) | 198941007 | Hypertension complicating pregnancy, childbirth and the puerperium (disorder) | 1 | 1 |
| 428809009 | Repair of aorto-uniiliac aneurysm with insertion of endovascular stent graft (procedure) | 233407007 | Insertion of abdominal aorta stent (procedure) | 1 | 1 |
| 174899009 | Repair of sinus venosus (procedure) | 119765004 | Heart closure (procedure) | 1 | 1 |

| | | | | | |
|---|---|---|---|---|---|
| 735579001 | Residual interatrial communication following procedure (disorder) | 449351005 | Residual interatrial communication (disorder) | 1 | 1 |
| 23648008 | Restoration, crown, resin with high noble metal (procedure) | 27121007 | Restoration, crown, resin with noble metal (procedure) | 1 | 1 |
| 232069006 | Retinal pigment epithelial detachment with tear of retinal pigment epithelium (disorder) | 95690009 | Retinal tear (disorder) | 0.999999891 | 0.999982589 |
| 15699201000119100 | Retinitis pigmentosa of left eye (disorder) | 399463004 | Retinitis (disorder) | 1 | 1 |
| 4410001 | Retroperitoneal hernia with obstruction (disorder) | 280130008 | Disorder of soft tissue of abdominal cavity (disorder) | 1 | 1 |
| 412766004 | Revision of dacryocystorhinostomy and insertion of tube (procedure) | 609319000 | Revision of dacryocystorhinostomy (procedure) | 1 | 1 |
| 231556003 | Revision of orbital floor implant (procedure) | 29358007 | Revision of orbital implant (procedure) | 1 | 1 |
| 450815006 | Revision of prosthesis of abdominal aorta (procedure) | 405369007 | Revision of abdominal vascular prosthesis (procedure) | 1 | 1 |
| 175332001 | Revision of prosthesis of bifurcation of aorta (procedure) | 450815006 | Revision of prosthesis of abdominal aorta (procedure) | 1 | 1 |

| | | | | | |
|---|---|---|---|---|---|
| 461629004 | Right aortic arch branching pattern (disorder) | 111321007 | Right aortic arch (disorder) | 1 | 1 |
| 461629004 | Right aortic arch branching pattern (disorder) | 111321007 | Right aortic arch (disorder) | 1 | 1 |
| 1076571000119100 | Scapulothoracic bursitis of left shoulder (disorder) | 303111000119104 | Bursitis of left shoulder (disorder) | 1 | 1 |
| 15737361000119100 | Secondary cataract of right eye (disorder) | 766834007 | Secondary cataract (disorder) | 1 | 1 |
| 94393006 | Secondary malignant neoplasm of lymph nodes of face (disorder) | 94293008 | Secondary malignant neoplasm of face (disorder) | 0.999999781 | 0.982854456 |
| 188454009 | Secondary malignant neoplasm of skin of head (disorder) | 94586008 | Secondary malignant neoplasm of soft tissues of head (disorder) | 1 | 1 |
| 305682005 | Seen by medical ophthalmologist (finding) | 305721007 | Seen by ophthalmologist (finding) | 0.999999892 | 0.990906356 |
| 248118000 | Self-induced purging to lose weight (disorder) | 249520001 | Self-induced purging (disorder) | 0.999999923 | 0.999991408 |
| 789585000 | Sensory polyneuropathy due to diabetes mellitus (disorder) | 127011001 | Sensory neuropathy due to diabetes mellitus (disorder) | 0.999999287 | 0.99506456 |
| 371599001 | Severe bipolar I disorder (disorder) | 371596008 | Bipolar I disorder (disorder) | 0.99999982 | 0.999986711 |
| 18242003 | Shortening of eyelid margin (procedure) | 2242005 | Reconstruction of eyelid (procedure) | 1 | 1 |
| 87152003 | Soft tissue release with peroneus brevis tendon transfer (procedure) | 264991006 | Transfer of peroneus brevis (procedure) | 1 | 1 |

| | | | | | |
|---|---|---|---|---|---|
| 723162005 | Spastic monoplegia of lower limb (disorder) | 386781001 | Spastic syndrome (disorder) | 0.99999984 | 0.999941242 |
| 8847002 | Spondylosis (disorder) | 371082009 | Arthritis of spine (disorder) | 0.972420888 | 0.926143981 |
| 46860007 | Sprain of ligament of cricothyroid joint (disorder) | 105611005 | Sprain of ligament of joint (disorder) | 1 | 1 |
| 105121000119102 | Squamous cell carcinoma of vagina (disorder) | 254893005 | Carcinoma of vagina (disorder) | 0.999999651 | 0.983428201 |
| 254661000 | Squamous cell papilloma of skin (disorder) | 255184001 | Papilloma of skin (disorder) | 0.999999845 | 0.997211618 |
| 331961000119108 | Strabismic amblyopia of right eye (disorder) | 336451000119108 | Amblyopia of right eye (disorder) | 0.999999572 | 0.992858145 |
| 726194009 | Strain of fascia of intrinsic muscle of thumb (disorder) | 726191001 | Injury of fascia of intrinsic muscle of thumb (disorder) | 1 | 1 |
| 446198006 | Strain of infraspinatus muscle (disorder) | 239954007 | Soft tissue lesion of shoulder region (disorder) | 0.999999749 | 0.999995698 |
| 11314321000119100 | Stress fracture of finger of left hand (disorder) | 704066009 | Stress fracture of phalanx of finger (disorder) | 1 | 1 |
| 11314281000119100 | Stress fracture of finger of right hand (disorder) | 704066009 | Stress fracture of phalanx of finger (disorder) | 1 | 1 |
| 123609007 | Subacute glomerulonephritis (disorder) | 19342008 | Subacute disease (disorder) | 1 | 1 |

| | | | | | |
|---|---|---|---|---|---|
| 198102006 | Subacute non-puerperal mastitis (disorder) | 70912006 | Subacute mastitis (disorder) | 1 | 1 |
| 446563000 | Subchondral hematoma of pinna (disorder) | 281464006 | Subchondral hematoma (disorder) | 0.999999878 | 0.999991195 |
| 11772691000119100 | Subluxation of radial head of left elbow (disorder) | 417109008 | Subluxation of radial head (disorder) | 1 | 1 |
| 11772651000119100 | Subluxation of radial head of right elbow (disorder) | 417109008 | Subluxation of radial head (disorder) | 1 | 1 |
| 304594002 | Suicidal intent (finding) | 267073005 | Suicidal (finding) | 0.999978845 | 0.999020757 |
| 40194002 | Superficial injury of lip without infection (disorder) | 11847211000119100 | Superficial injury of lip (disorder) | 0.999998776 | 0.999844274 |
| 446620009 | Superficial laceration of shoulder (disorder) | 239954007 | Soft tissue lesion of shoulder region (disorder) | 1 | 1 |
| 385005009 | Surgical lateral margin involved by malignant neoplasm (finding) | 372263007 | Surgical lateral margin involved by tumor (finding) | 1 | 1 |
| 15438004 | Suture of facial nerve, extracranial (procedure) | 3666002 | Peripheral neurorrhaphy (procedure) | 1 | 1 |
| 7418001 | Suture of fascia to skeletal attachment of hand (procedure) | 66342006 | Repair of fascia of hand (procedure) | 1 | 1 |
| 427681009 | Synovial cyst of sacrum (disorder) | 280136002 | Disorder of soft tissue of lower limb (disorder) | 0.999999982 | 0.999999952 |

| | | | | | |
|---|---|---|---|---|---|
| 10759921000119100 | Syphilis in mother complicating childbirth (disorder) | 76272004 | Syphilis (disorder) | 0.999997453 | 0.999976169 |
| 38523005 | Syphilitic parkinsonism (disorder) | 230182006 | Late syphilitic encephalitis (disorder) | 0.999999967 | 0.999997626 |
| 91567001 | Take-down of stoma of bronchus (procedure) | 72558007 | Closure of stoma of thorax (procedure) | 1 | 1 |
| 15668201000119100 | Talipes varus of left foot (disorder) | 766243008 | Talipes varus (disorder) | 1 | 1 |
| 15674961000119100 | Tendinitis of bilateral hips (disorder) | 34840004 | Tendinitis (disorder) | 1 | 1 |
| 298168007 | Thumb joint inflamed (finding) | 298167002 | Finger joint inflamed (finding) | 0.999999125 | 0.969989225 |
| 237506002 | Thyrotoxicosis in pregnancy (disorder) | 72271000119100 | Hyperthyroidism in pregnancy (disorder) | 0.999999874 | 0.985 |
| 16006591000119100 | Tophus of left hand co-occurrent and due to gout (disorder) | 190842000 | Tophus of hand co-occurrent and due to gout (disorder) | 1 | 1 |
| 16006551000119100 | Tophus of right hand co-occurrent and due to gout (disorder) | 190842000 | Tophus of hand co-occurrent and due to gout (disorder) | 1 | 1 |
| 472916000 | Toxic metabolic encephalopathy (disorder) | 28394000 | Toxic encephalopathy (disorder) | 0.997847975 | 0.95371643 |
| 237279007 | Transient hypertension of pregnancy (disorder) | 52698002 | Transient hypertension (disorder) | 0.999213493 | 0.87365673 |
| 276558007 | Transient neonatal hyperglycemia (disorder) | 237623001 | Acute hyperglycemia (disorder) | 1 | 1 |
| 331791000119103 | Transient visual loss of right eye (disorder) | 456161000124109 | Disorder of right eye (disorder) | 0.999999888 | 0.999997696 |

| | | | | | |
|---|---|---|---|---|---|
| 58324005 | Transplantation of dura (procedure) | 2315006 | Operation on cerebral meninges (procedure) | 1 | 1 |
| 388229009 | Transurethral external sphincterotomy of male bladder (procedure) | 388228001 | Transurethral incision of bladder neck (procedure) | 1 | 1 |
| 109669008 | Traumatic arthritis of the temporomandibular joint (disorder) | 58188004 | Traumatic arthropathy (disorder) | 1 | 1 |
| 400184006 | Traumatic blister of trunk (disorder) | 280133005 | Disorder of soft tissue of trunk (disorder) | 0.999996265 | 0.99999038 |
| 271131005 | Traumatic blister of trunk, infected (disorder) | 373592004 | Traumatic blister (disorder) | 0.999996273 | 0.999583386 |
| 262760009 | Traumatic dislocation of ossicular prosthesis (disorder) | 232265009 | Traumatic ossicular dislocation (disorder) | 1 | 1 |
| 16899621000119100 | Traumatic perforation of rectum (disorder) | 50008003 | Traumatic perforation of large intestine (disorder) | 1 | 1 |
| 396979005 | Tumor invades optic nerve at lamina cribrosa (finding) | 399573004 | Tumor invades optic nerve (finding) | 1 | 1 |
| 282838005 | Unable to move from a position (finding) | 225601007 | Unable to move (finding) | 1 | 1 |
| 305492009 | Under care of medical ophthalmologist (finding) | 305544007 | Under care of ophthalmologist (finding) | 1 | 1 |
| 25696006 | Venereal Disease Research Laboratory, qualitative (procedure) | 82191002 | Syphilis test, qualitative (procedure) | 1 | 1 |

| | | | | | |
|---|---|---|---|---|---|
| 446784007 | Venography of lower extremity using contrast (procedure) | 446030004 | Angiography using contrast (procedure) | 0.99999992 | 0.995885312 |
| 4381006 | Verbal paraphasia (finding) | 53096005 | Paraphasia (finding) | 0.999999925 | 0.999994532 |
| 219396005 | War injury due to explosion of sea-based artillery shell (disorder) | 219403007 | War injury due to explosion of artillery shell (disorder) | 1 | 1 |
| 219397001 | War injury due to explosion of torpedo (disorder) | 274230000 | War injury (disorder) | 0.999999852 | 0.999982818 |
| 444412001 | Willing to be donor of liver (finding) | 225466006 | Willing (finding) | 1 | 1 |
| 54736008 | Xanthoma planum of eyelid (disorder) | 6400008 | Xanthoma of eyelid (disorder) | 1 | 1 |
