# Supplementary material for: Quantitatively assessing the impact of the quality of SNOMED CT subtype hierarchy on cohort queries
Source: J Am Med Inform Assoc. 2024 Nov 9;32(1):89–96. doi: 10.1093/jamia/ocae272 (PMC11648736; doi:10.1093/jamia/ocae272)
Supplement: ocae272_Supplementary_Data [file ocae272_supplementary_data.zip › Suppl/inaccurate-relations-metrics.pdf]

subconcept id	subconcept	superconcept id	superconcept	micro-averaged precision	macro-averaged precision
722410009	Somatic dysfunction of lumbosacral region (finding)	718936004	Somatic dysfunction of sacral region (finding)	0.999999229	0.999954694
253190004	Lumbosacral agenesis (disorder)	205425003	Sacral agenesis (disorder)	NA	NA
301227004	Tracheobronchial finding (finding)	301229001	Bronchial finding (finding)	0.994909493	0.989714021
276543004	Tracheobronchial hemorrhage (disorder)	405541003	Bronchial hemorrhage (disorder)	0.999999689	0.957142357
276543004	Tracheobronchial hemorrhage (disorder)	233783005	Tracheal hemorrhage (disorder)	0.999999564	0.946423419
88366003	Reconstruction of mandible (procedure)	89643001	Osteoplasty of mandible (procedure)	NA	NA
39834009	Nephrostomy (procedure)	88734005	Renal pyelostomy (procedure)	0.999966999	0.931382254
187865009	Malignant neoplasm of middle lobe bronchus (disorder)	126710000	Neoplasm of bronchus of right middle lobe (disorder)	0.999999945	0.98749392
24518007	Closure of laryngotracheal fistula (procedure)	27660008	Closure of laryngeal fistula (procedure)	NA	NA
106133000	Language finding (finding)	106132005	Speech finding (finding)	0.995091089	0.935850128
767575003	Functional communication training (procedure)	310829009	Communication skills training (procedure)	NA	NA
239289001	Fixation of fracture using plate (procedure)	257835007	Internal fixation using plate (procedure)	0.999998778	0.99675936
225968000	Liaising with relative (procedure)	711066004	Liaising with family (procedure)	NA	NA
248692001	Mid-systolic click (finding)	3747008	Ejection click (finding)	0.999999459	0.964404762

192136006	Specific reading disorder (disorder)	52824009	Developmental reading disorder (disorder)	0.999859085	0.930415802
254780006	Arteriovenous malformation of skin (disorder)	234141001	Congenital arteriovenous malformation (disorder)	1	1
253800006	Complete duplication of appendix (disorder)	35266001	Congenital duplication of appendix (disorder)	NA	NA
231576009	Repair of medial canthus (procedure)	27491009	Medial canthoplasty (procedure)	NA	NA
175612005	Percutaneous transluminal angioplasty of iliac artery (procedure)	20500004	Percutaneous transluminal iliac artery balloon angioplasty (procedure)	NA	NA
287364001	Arteriovenous anastomosis (procedure)	123679006	Arterial anastomosis (procedure)	0.999987652	0.993391097
299970006	Animal sting (disorder)	371058004	Venomous sting (disorder)	0.999999883	0.999959924
90686009	Angiography of arteries of bilateral extremities (procedure)	53495004	Angiography of arteries of bilateral upper extremities (procedure)	NA	NA
50122000	Metabolic encephalopathy (disorder)	472916000	Toxic metabolic encephalopathy (disorder)	0.997386857	0.949386344
47435007	Administration of vaccine product containing only Measles morbillivirus antigen (procedure)	572481000119103	Administration of vaccine product containing only live attenuated Measles morbillivirus antigen (procedure)	0.999999494	0.999753078
28394000	Toxic encephalopathy (disorder)	472916000	Toxic metabolic encephalopathy (disorder)	0.998837262	0.983344707
15631451000119100	Intermittent exotropia of right eye (disorder)	334731000119104	Intermittent monocular exotropia of right eye (disorder)	NA	NA

15631491000119100	Intermittent exotropia of left eye (disorder)	340331000119109	Intermittent monocular exotropia of left eye (disorder)	NA	NA
292064003	Pethidine adverse reaction (disorder)	292061006	Pethidine analog adverse reaction (disorder)	NA	NA
295197009	Pethidine overdose (disorder)	295188005	Pethidine analog overdose (disorder)	NA	NA
293606006	Allergy to pethidine (finding)	293603003	Allergy to pethidine analog (finding)	0.999999882	0.998507233
240179004	Pelvis juvenile osteochondropathy (disorder)	240180001	Hip juvenile osteochondropathy (disorder)	NA	NA
193434008	Focal juxtapapillary choroiditis (disorder)	193436005	Juxtapapillary focal chorioretinitis (disorder)	NA	NA
267073005	Suicidal (finding)	6471006	Suicidal thoughts (finding)	0.999963966	0.999701918
92564006	Carcinoma in situ of uterine cervix (disorder)	406103009	Squamous cell carcinoma in situ of uterine cervix (disorder)	0.99826115	0.9296958
451000119106	Closed injury of head (disorder)	430937009	Closed wound of head (disorder)	0.999695677	0.996943644
698077007	Compression fracture of thoracic spine (disorder)	5301000124104	Compression fracture of thoracic vertebra (disorder)	0.999627986	0.971627106
35246005	Anorectal cellulitis (disorder)	84681008	Rectal cellulitis (disorder)	0.999999981	0.997865632
72779005	Anorectal fistula (disorder)	80736008	Rectal fistula (disorder)	0.99992625	0.982869519
403914000	Superficial basal cell carcinoma (disorder)	254701007	Basal cell carcinoma of skin (disorder)	1	1
713081000	Dissection of cerebral artery (disorder)	429221000124104	Dissecting aneurysm of cerebral artery (disorder)	0.99998294	0.961534612

35246005	Anorectal cellulitis (disorder)	41152008	Anal cellulitis (disorder)	0.999999966	0.996186395
33781009	Abdominopelvic abscess (disorder)	75100008	Abdominal abscess (disorder)	1	1
402786009	Chylomicronemia syndrome (disorder)	402726006	Primary chylomicronemia (disorder)	0.999999435	0.897368421
301227004	Tracheobronchial finding (finding)	301228009	Finding of trachea (finding)	0.993663588	0.862430158
83507006	Finding of thought content (finding)	55956009	Disturbance in content of thought (finding)	0.959861221	0.535810762
61090006	Crown, full cast high noble metal (procedure)	84703002	Restoration, crown, full cast, high noble metal (procedure)	NA	NA
72779005	Anorectal fistula (disorder)	786878009	Fistula of anus (disorder)	0.999993836	0.999761095
118746000	Procedure on shoulder joint (procedure)	699464003	Procedure on joint of shoulder girdle (procedure)	0.999865168	0.828953523
